# Deciphering the Unconventional Reduction of C=N Bonds by Old Yellow Enzymes Using QM/MM

**DOI:** 10.1021/acscatal.3c04362

**Published:** 2024-01-10

**Authors:** Amit Singh Sahrawat, Nakia Polidori, Wolfgang Kroutil, Karl Gruber

**Affiliations:** † Institute of Molecular Biosciences, University of Graz, Graz 8010, Austria; ‡ Institute of Chemistry, University of Graz, Graz 8010, Austria; § Field of Excellence BioHealth, University of Graz, Graz 8010, Austria; ∥ BioTechMed-Graz, Graz 8010, Austria

**Keywords:** oxidoreductase, flavin, enzyme mechanism, computational modeling, natural bond orbital analysis

## Abstract

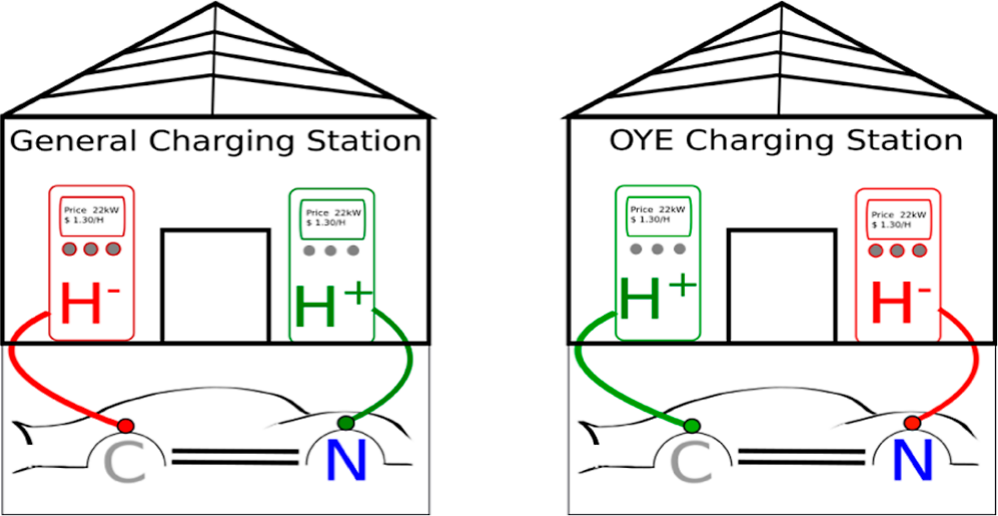

The reduction of C=X (X = N, O) bonds is a cornerstone in both synthetic organic chemistry and biocatalysis. Conventional reduction mechanisms usually involve a hydride ion targeting the less electronegative carbon atom. In a departure from this paradigm, our investigation into Old Yellow Enzymes (OYEs) reveals a mechanism involving transfer of hydride to the formally more electronegative nitrogen atom within a C=N bond. Beyond their known ability to reduce electronically activated C=C double bonds, e.g., in α, β-unsaturated ketones, these enzymes have recently been shown to reduce α-oximo-β-ketoesters to the corresponding amines. It has been proposed that this transformation involves two successive reduction steps and proceeds via imine intermediates formed by the reductive dehydration of the oxime moieties. We employ advanced quantum mechanics/molecular mechanics (QM/MM) simulations, enriched by a two-tiered approach incorporating QM/MM (UB3LYP-6-31G*/OPLS2005) geometry optimization, QM/MM (B3LYP-6-31G*/amberff19sb) steered molecular dynamics simulations, and detailed natural-bond-orbital analyses to decipher the unconventional hydride transfer to nitrogen in both reduction steps and to delineate the role of active site residues as well as of substituents present in the substrates. Our computational results confirm the proposed mechanism and agree well with experimental mutagenesis and enzyme kinetics data. According to our model, the catalysis of OYE involves hydride transfer from the flavin cofactor to the nitrogen atom in oximoketoesters as well as iminoketoesters followed by protonation at the adjacent oxygen or carbon atoms by conserved tyrosine residues and active site water molecules. Two histidine residues play a key role in the polarization and activation of the C=N bond, and conformational changes of the substrate observed along the reaction coordinate underline the crucial importance of dynamic electron delocalization for efficient catalysis.

## Introduction

**Scheme 1:**
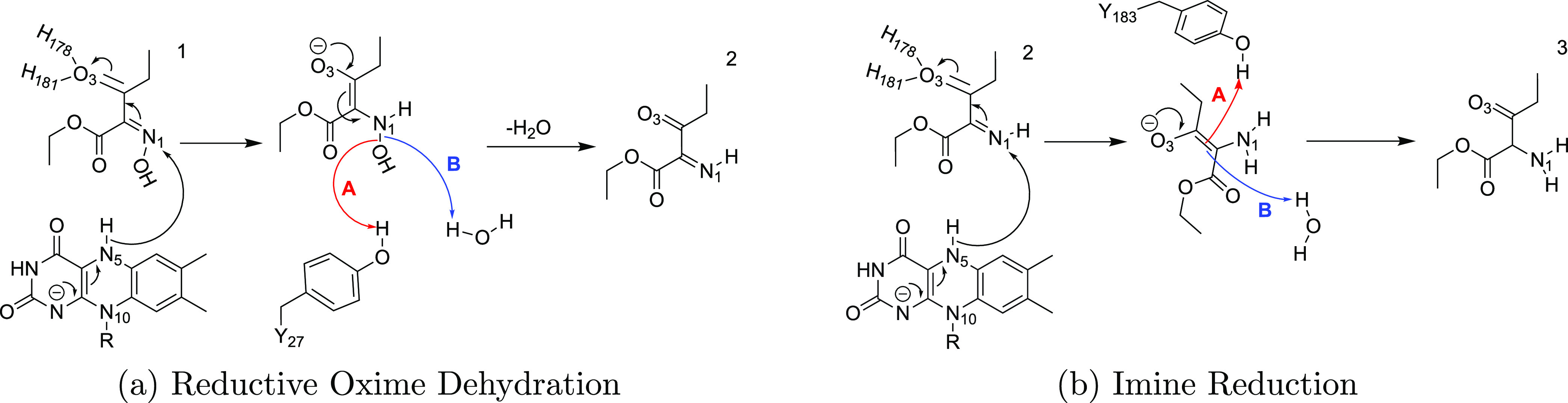
Proposed Mechanism for the Ene-Reductase-Mediated Conversion of α-Oximo-β-ketoester **1** to the Corresponding α-Amino-β-ketoester **3** Via an α-Imine **2** Intermediate A and B denote the possibility of direct proton transfer from the respective enzyme residue or the nearest solvent molecule, respectively. The scheme depicts the active site of XenA.

The reduction of C=X double bonds (with X = N or O) represents a fundamental and widely used transformation in synthetic chemistry and biocatalysis.^1−5^ It provides an indispensable route to a broad range of complex organic compounds and, therefore, has wide-ranging applications in the synthesis of pharmaceuticals and agrochemicals, in environmental remediation and beyond.^6−14^ Conventionally, the reduction of C=X bonds proceeds through the transfer of a hydride ion to the less electronegative carbon atom, followed by a proton transfer to the more electronegative heteroatom X. This fundamental mechanistic understanding has played a significant role in shaping many synthetic and biocatalytic methods.^15−26^ However, ene-reductases from the Old Yellow Enzyme (OYE) family offer a distinct perspective on this conventional understanding. Widely used for the asymmetric reduction of electronically activated C=C double bonds, e.g., in α, β-unsaturated ketones,^25,27−36^ these enzymes have also recently been demonstrated to catalyze the reduction of α-oximo β-ketoesters to the respective α-amino compounds via α-imine intermediates (as shown in Scheme 1 for the conversion of ethyl-(*Z*)-2-(hydroxyimino)-3-oxopentanoate **1**).^37,38^ This OYE-catalyzed transformation is assumed to involve two “ping-pong” cycles.^29,31,39^ The first phase involves the conversion of **1** to the respective α-imine intermediate **2** via hydride transfer and a dehydration reaction (Scheme 1a). Similarly, in the second cycle, **2** is reduced to an amine **3** via accepting a hydride from the flavin, followed by proton transfer from the solvent or the enzyme itself (Scheme 1b). Although the broad outlines of these “ping-pong” cycles are well-established, one of the most intriguing aspects within this proposed mechanism is the unconventional hydride transfer to the more electronegative nitrogen atom within the C=N double bond of **1** and **2**. This defies the conventional understanding that a hydride should approach a less electronegative carbon atom. While our previous study provided essential chemical and structural details to support the proposed mechanism,^38^ we are here employing hybrid quantum mechanics/molecular mechanics (QM/MM) computations to decipher further mechanistic intricacies unattainable by experimental approaches. Our QM/MM approach involves a two-tier implementation to reveal the most refined details of the underlying reaction mechanism. At first, we focus more on the structural level pertaining to the mechanistic details of each reaction coordinate along the proposed reaction pathway. This task was achieved by using “static” techniques like QM/MM geometry optimization. Furthermore, to discover the electronic and energetic significances behind each reaction, we have chosen the more accurate and dynamic approach like QM/MM steered molecular dynamics (SMD) simulations coupled with natural bond orbital (NBO)^40^ analysis. Overall, the present work demonstrates how an ene-reductase from the OYE family redefines the reduction of C=N double bonds through an unconventional approach. By capitalizing on the existing delocalized electron density of its atypical substrate, the enzyme showcases its remarkable adaptability and sheds new light on the intricacies of this biotransformation.

## Results and Discussion

The starting point of our calculations was the crystal structure of the complex of XenA from *Pseudomonas putida* with **1**.^38^ The crystal structure then went through preprocessing steps, majorly on the two active site histidines H178 and H181, which were protonated on the ϵ and δ nitrogen atoms, respectively (Figure S1a). Also, the flavin was modeled in its reduced state (FMNH^–^). Subsequently, the prepared structure went through solvation, neutralization, MM minimization, and MM equilibration, followed by QM/MM energy minimization and a 5 ps QM/MM equilibration run. We refer to the last frame of this equilibration run as QM/MM equilibrated structure for **1** (S_eq_^1^). From here, S_eq_^1^ was subjected to our two-tier methodology to gain comprehensive insights into the reaction pathway and energy barriers. The two methodologies, namely QM/MM geometry optimization and QM/MM SMD simulations, were thoughtfully chosen based on their respective strengths and weaknesses in characterizing molecular structures along the reaction coordinate and computing accurate energy barriers.^41−43^ QM/MM geometry optimization technique leverages advanced algorithms to determine the molecular structures at stationary points along the reaction pathway, yielding precise geometrical information.^44−46^ However, it has limitations in accurately estimating the energy barrier for the reaction pathway due to inadequate sampling. In contrast, QM/MM SMD simulations employ sophisticated sampling techniques, enabling a more comprehensive exploration of the conformational spaces and transition states relevant to a reaction pathway, ultimately leading to improved accuracy in energy barrier estimation.^41−43^ After performing the recommended evaluations,^47,48^ we have chosen a QM system comprising **1**/**2**, lumiflavin (LuF), side chains of Y27, H178, H181, and Y183 residues, and the nearest water molecule. Overall, a total of 100 independent 1 ps QM/MM SMD simulations were performed for each reaction pathway. The whole scheme of our computational approach is summarized in Scheme S1 and is described in detail in the Supporting Information.

### Reductive Oxime Dehydration

#### Structural Analysis of Binding Pose

We took the S_eq_^1^ and optimized it using (UB3LYP:6-31G*/OPLS4 ff) computations and, from here on, refer to it as QM/MM optimized structure for **1** (S_opt_^1^). The binding poses of substrate **1** in the crystal structure and S_opt_^1^ are similar. However, there are very fine differences across the distance and angle pertaining to the hydride transfer reaction. For example, the distance between the N1 atom of **1** and the flavin’s N5 is 2.9 Å in S_opt_^1^, compared to 3.7 Å in the crystal structure. Furthermore, the angle defined by the flavin N10, the flavin N5, and the substrate N1 atom is 70.6 deg in the crystal structure, while the same is measured at 103.1 deg in S_opt_^1^. The structural superimposition of the crystal structure and S_opt_^1^ is shown in Figure S1b. In our understanding, these marginal differences persist because of the different oxidation states of the flavin cofactor; the crystal structure contains oxidized flavin (FMN), whereas we have considered the reduced flavin (FMNH^–^), represented by the LuF in our QM/MM computation. Nevertheless, S_opt_^1^ has characteristic binding features similar to typical OYE substrates,^28,33^ e.g., the carbonyl oxygen (O3) has two hydrogen bonds with H178 and H181 (Figure S1c), the hydride receiving atom (N1) is within 3.5 Å of flavins’ N5 atom, and the vectors of N10–N5 and N5–N1 make an obtuse angle at their intersect. Along with these fixed values of the aforementioned geometrical parameters, we also obtained their respective statistical summary by averaging three independent 5 ps QM/MM production runs on the S_eq_^1^ (Table S1). The polar interactions between the substrate **1** and the active site residues have been summarized in Figure S1c.

### Reaction Mechanism

#### Hydride Transfer Occurs First, Followed by Proton Transfer

**Figure 1:**
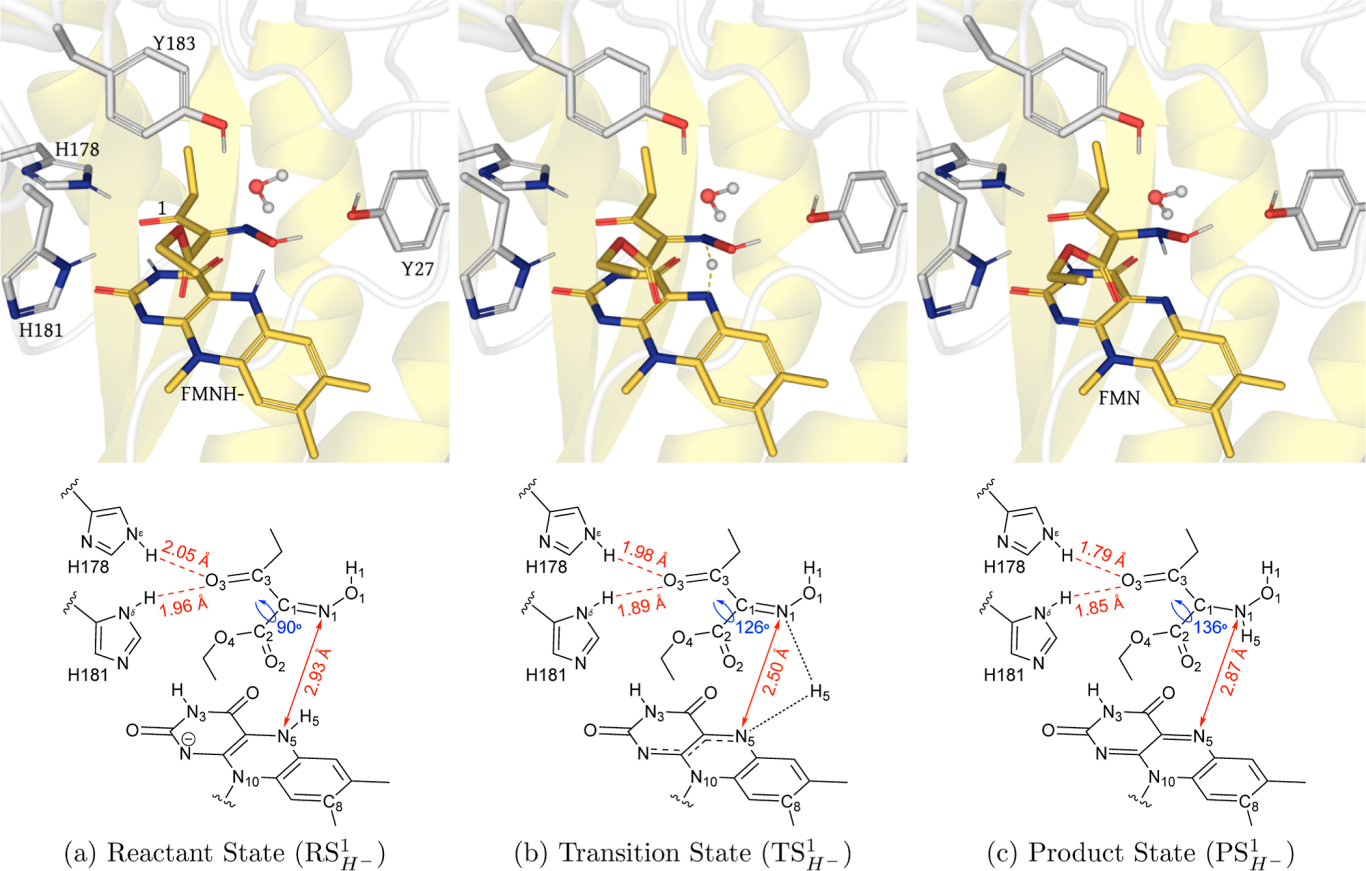
(Top) QM/MM (UB3LYP-6-31G*/OPLS2005) geometry optimized structures of the active site of XenA in the (),(), and () configurations with **1** during the hydride transfer. The substrate **1** and LuF are shown in yellow sticks, side chains of active site residues are shown in light gray sticks, and water is displayed in the ball and stick representation. In (), the transient H5 atom is also depicted as a gray ball. (Bottom) Schematic of each configuration showing key distances (red) and dihedrals (blue).

Conventionally, the inherent polarization of the C=N double bond readies it for a hydride transfer to the partially positive C atom, followed by a proton transfer to the partially negative N atom, as demonstrated by imine reductases.^21,22^ Despite this, imine reductases failed to reduce **1** and similar substrates.^37,49^ It is plausible that the carbonyl groups attached to the C=N double bond might have altered its innate polarity, thereby obstructing its conventional reduction by imine reductases. Remarkably, the presence of conjugated double bonds along the O3=C3–C1=N1 part of **1** is similar to the typical OYE substrates like in α, β-unsaturated ketones (O=C–Cα=Cβ), where the electron-withdrawing carbonyl group anchored to the active site, activating the Cα=Cβ double bond for hydride abstraction via partial polarization. Similarly, substrate **1** features two electron-withdrawing carbonyl groups attached to the C1=N1 unsaturation. However, in the active site, the C2=O2 group does not lie on the same plane as the C1=N1 bond, potentially limiting its electron-withdrawing capability (Figure 1a). Conversely, the C3=O3 group, being planar with the C1=N1 bond, may serve as a more effective electron-withdrawing group. Overall, in the context of the active site of the OYE, the N1 atom of substrate **1** has similar stereochemical features to Cβ in (O=C–Cα=Cβ) and hence is staged as a potential hydride acceptor. Following this resemblance, the N1 atom in substrate **1** is proposed to receive a hydride from the reduced flavin, and the O1 atom is set to receive a proton from the Y27 or a nearby water molecule (Figure 1a). Overall, the proposed mechanism yields an imine; however, it is not known whether the proton and the hydride are added together or as distinct steps. Using S_opt_^1^ as starting structure, a two-dimensional (2D) potential energy surface was generated (Figure S2) with the hydride and proton transfer reaction coordinates ( and ) varied simultaneously to determine the order of events. It turns out that substrate **1** undergoes a two-step reaction; the initial step is hydride transfer, followed by proton-mediated dehydration.

### Hydride Transfer Step

#### Structural Analysis of Reaction Coordinates

Optimized molecular structures corresponding to stationary points along the lowest energy reaction coordinate for hydride transfer are depicted in Figure 1, mentioning the key geometrical parameters. We found that the distance between the H5 atom and acceptor N1 decreases from 2.1 Å in  to 1.2 Å in  and then to 1.0 Å in . Whereas the distance between the hydride donor (N5) and hydride acceptor (N1) decreases from 2.93 Å in  to 2.50 Å in  and then increases to 2.87 Å in . In contrast, the angle between the N5–H5–N1 approaches linearity, from 137° in  to 159° in  and from there to , it remains largely unchanged. Additionally, the distances between the O3 and the polar hydrogen atoms of the two histidine side chains (H178 and H181) are shorter for  and  compared to , likely stabilizing the negative charge forming on the oxygen atom during the hydride transfer.^28,33^ H181 is relatively closer to O3, whereas H178 shortens its distance from O3 in the end of the reaction (Figure S3a). The contrasting variability across C1–N1, C1–C3, and C3–O3 bonds indicates charge delocalization (Figure S3b). While the electron density of π(C1=N1) delocalized over the neighboring bonds located in the same plane, O3 is the foremost beneficiary of this delocalization due to its higher electronegativity and its proximity to the active site histidines. Interestingly, the influence of the delocalization of the negative charge can be seen over other parts of the substrate **1** as well, especially for the dihedral angle (C3–C1–C2–O2), which approaches planarity from 90° in  to 126° in  and finally reaches to 136° in . We postulate that this increase could facilitate a better orbital overlap between π(C1=N1) and the π*(C2=O2). Similarly, for better resonance sharing among N5 and its neighboring atoms, the butterfly twist of the isoalloxazine ring (measured by the changes in dihedral N3–N10–N5–C8) decreased as the hydride departed from the flavin (Figure S3c). Apart from these general parameters for OYE catalysis, the transition state geometries of nucleophilic additions can be explained by the Bürgi-Dunitz angle, which is a more specific parameter to define the optimal angle for an approaching nucleophile to a trigonal unsaturated electrophile.^50,51^ Ideally, for a maximum overlap between the nucleophile’s highest occupied molecular orbital and the electrophile’s lowest unoccupied molecular orbital, the Bürgi-Dunitz angle should be more than 90°. We also found that in , the hydride is approaching the C1=N1 bond at an angle of 111.2°, which is very similar to the previously reported Bürgi-Dunitz angles for ene-reductase mediated hydride addition to the C=C in cyclohexenone.^52^ The dynamic evolution of the geometric parameters discussed here is also monitored through QM/MM SMD simulations and is presented in Figure S3. The calculated activation energy (Δ*E*_a_^‡^) for the hydride transfer is 14.19 kcal/mol, whereas the computed free energy (Δ*G*^‡^) change for the same is 9.82 kcal/mol (Figure S4). In our previous work, the presteady-state rate for the reoxidation of flavin by the substrate **1** (*k*_ox_) was estimated to be 28.7 ± 3.2 s^–1^.^38^ Applying classical transition-state theory using the Eyring equation, this corresponds to a Δ*E*_a_^‡^ of 15.4 kcal/mol. Therefore, the calculated Δ*E*_a_^‡^ value for the hydride transfer seems reasonable. The computed enthalpy of the hydride transfer reaction is −0.44 kcal/mol.

### NBO Analysis of Reaction Coordinates

**Figure 2:**
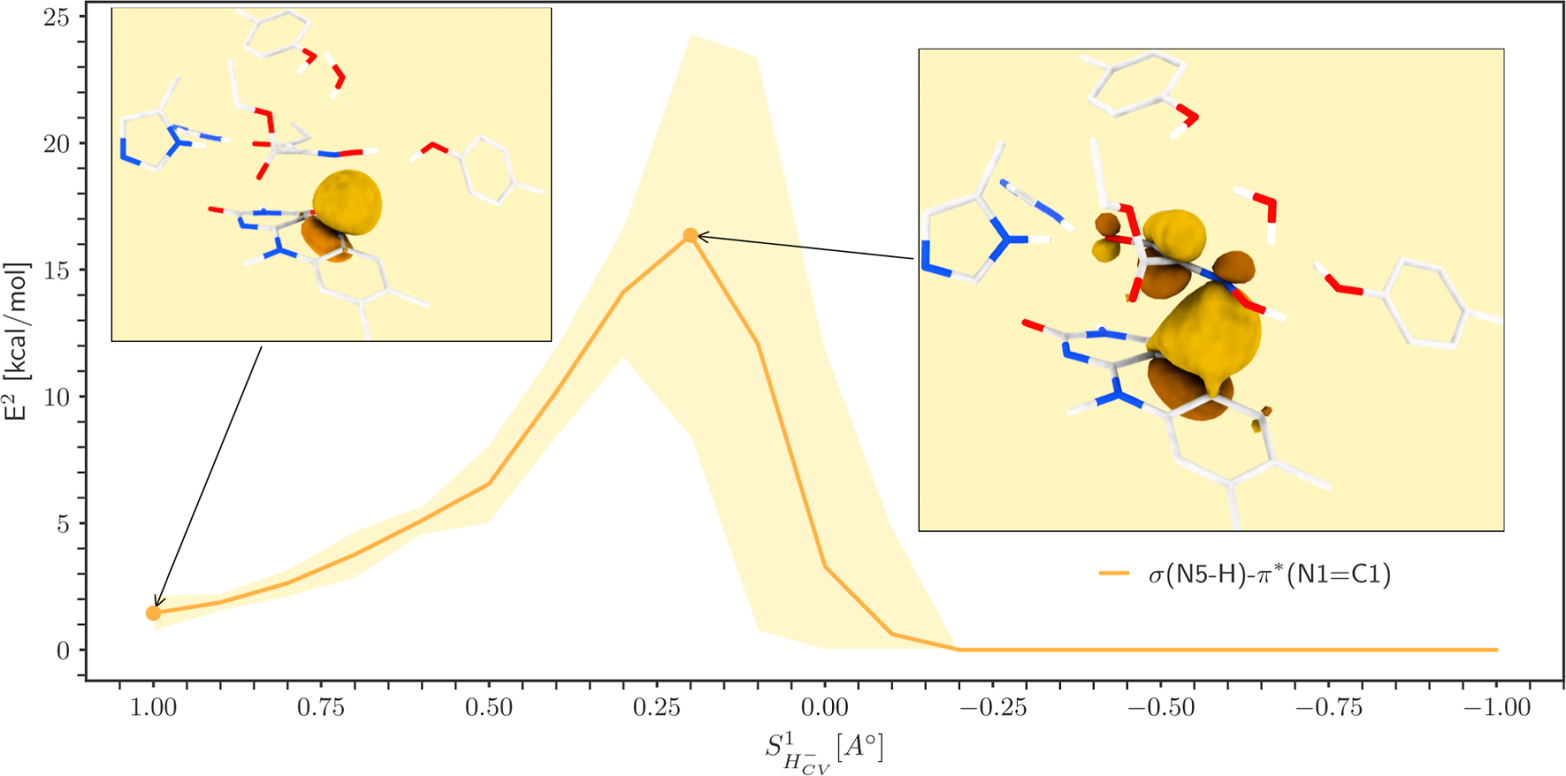
NBO Analysis of the Reaction Coordinates. Delocalization energy (*E*^2^) plot between the donor σ(N5–H5) and acceptor π*(C1=N1) NBOs. The standard deviations are shown as semitransparent bands, whereas the inset figures show the extent of σ(N5–H5) donor NBO’s perturbation due to delocalization.

The NBO analysis suggests a strong charge transfer interaction between the donor σ(N5–H5) and the acceptor π*(C1=N1) NBOs, which is evident from the rise of delocalization energy (*E*^2^) between them near the transition state (Figure 2). NBO’s *E*^2^ measures the strength of delocalization between the donor’s occupied NBOs, e.g., bonding (BD) or lone pairs (LP), and acceptor’s unoccupied NBOs, e.g., antibonding (BD*). The larger the *E*^2^ value is, the stronger the interaction between electron donor and acceptor NBOs, and consequently, the greater the extent of delocalization.^53−55^ Qualitatively, natural localized molecular orbital can be used to display the extent of corresponding donor NBO’s perturbation due to delocalization,^56^ which is shown in insets of Figure 2. The electron density of the donor σ(N5–H5) NBO is mostly delocalized over the N1, C1, C3, and the O3 atom, which is also evident from the changes in atom-centric charges during the reaction (Figure S5). As speculated before, O3 withdraws more electrons and, as a result, is more negatively charged than its counterpart, O2. Therefore, the hydrogen bonding interactions from the H178 and H181 side chains directly point toward the lone-pair (LP) orbitals of the O3 atom to stabilize the accumulated negative charge on it (Figure S6a). Due to the closeness, the electron density from the O3 atom is delocalized more on H181 at the beginning of the reaction, whereas H178 contributed significantly to stabilize the accumulated electron density on O3 only at the end of the reaction (Figure S6b). The charge transfer interaction between the σ(N5–H5)-π*(C1=N1) pair of NBOs strongly relates to the overall reaction involving two distinct steps. In contrast, it could have been a single-step reaction if the receiving NBO was σ*(N1–O1), eventually resulting in a one-step hydride transfer coupled dehydration reaction. After the hydride transfer to the N1 atom, another possibility is the formation of hydroxylamine, as C1 gains considerable electron density to fetch a proton from the surroundings. However, neither NBO analysis nor our previous experimental report^38^ suggests this outcome. We found that the electron density from C1 is delocalized over the other neighboring atoms, namely, O2, O3, and C3. Second, O1 has shown a stronger charge transfer interaction with the water molecule (Figure S7), whereas C1 has no comparable interactions with the nearby proton donor. Overall, the hydride transfer step staged the O1 atom with a considerable gain of electron density, enough to attract a proton from the surroundings.

### Proton Transfer Step

#### Structural Analysis of Reaction Coordinates

**Figure 3:**
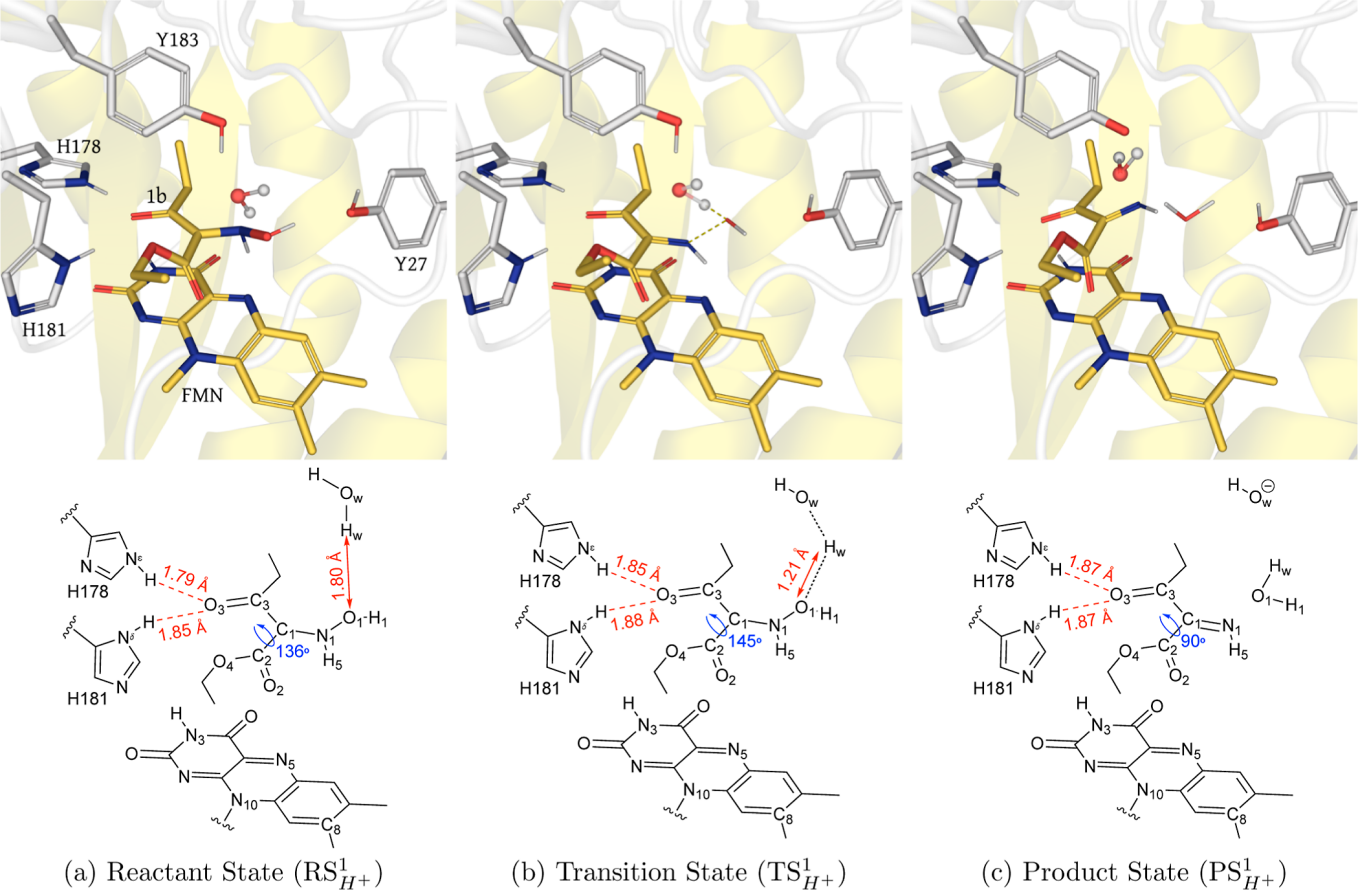
(Top) QM/MM (UB3LYP-6-31G*/OPLS2005) geometry optimized structures of the active site of XenA in the (), (), and () configurations with **1** during the proton transfer. The substrate **1** and LuF are shown in yellow sticks, side chains of active site residues are shown in light gray sticks, and water is displayed in the ball and stick representation. (Bottom) Schematic of each configuration showing key distances (red) and dihedrals (blue).

The addition of hydride to N1 slightly elongated the N1–O1 bond and moved the hydroxyl group out of the plane of the conjugated system (O3–C3–C1–N1). From here, O1 could fetch a proton from nearby water molecules or Y27; we have considered both possibilities and found that proton transfer from the water has a lower energy barrier (Figure S8). This is consistent with our previous experimental observation that the replacement of Y27 by phenylalanine did not significantly impact the overall reaction.^38^ Although the optimized molecular structures corresponding to stationary points along the lowest energy reaction coordinate for the direct proton transfer from Y27 are shown in (Figure S9), here we are considering water as a proton donor for the general case. The corresponding lowest energy reaction coordinates for direct proton transfer from water are shown in Figure 3. We used the product state of the hydride transfer step () as the reactant state () for the investigation of the proton transfer step. Interestingly, in the transition state of proton transfer (), the leaving substituent O1–H1 has a leaving angle of 113.7° with the C1=N1 bond, similar to the hydride approaching angle in  but in the opposite direction. As the proton approaches the O1 atom, the O1–H1 substituent becomes more and more bent out of the plane, which is pronounced by the decrease in C3–C1–N1–O1 dihedral angle from 153° in  to 116° in . Ow receives a proton from Y183 while donating a proton to the O1 atom; eventually, Y183 becomes an indirect proton donor. Consequently, the native proton dissociates from the water molecule, and as soon as it binds to the atom O1, another water molecule forms. Also, there is no longer an extra force exerted by the delocalization of electrons, which could hold the dihedral angle (C3–C1–C2–O2) closer to planarity as it approaches its original orthogonal position in  from 136° in , where the ester oxygen O4 has favorable polar interactions with the nearby solvent molecules. As soon as O1–H1 leaves the substrate **1**, the substrate’s atomic charges and bond lengths approach their original values at . Especially, the C1=N1 now regains its π electrons that are delocalized during hydride addition; similarly, the carbonyl (C3=O3) bond becomes shorter and less polarized. Eventually, this dehydration reaction completes the conversion of oxime **1** to imine **2**. The dynamic evolution of geometrical parameters discussed here is presented in (Figure S10). The calculated Δ*E*_a_^‡^ and Δ*G*^‡^ for the proton transfer from Y183 via water are 10.69 kcal/mol and 5.23 ± 0.12 kcal/mol, respectively. Whereas the computed enthalpy for the same is 0.26 kcal/mol.

### NBO Analysis of Reaction Coordinates

The occupancy of the lone-pair orbital of the O1 atom showed a considerable increase after the hydride transfer. Eventually, the O1 atom sought to delocalize the increased electron density on the nearby vacant nonbonding orbitals; as a result, O1 removes an H^+^ from the nearby polar protic solvent molecule, water. Simultaneously, Y183 also loses its proton to the same water molecule, donating a proton to O1. Overall, a proton was transferred to the O1 from Y183 via a water molecule. An analysis of the extent of E^2^ between respective donor–acceptor pairs of NBOs is shown in Figure S11. The dynamic changes in atom-centric charges during the proton transfer reaction are shown in Figure S12.

### Imine Reduction

#### Structural Analysis of Binding Pose

Revisiting the proposed ping-pong mechanism, where reductive dehydration of substrate **1** produces intermediate **2**, which exits the enzyme’s active site. Then, NADH enters the site, reduces the flavin molecule, and leaves the active site. Finally, intermediate **2** re-enters for the reduction of its C=N double bond. However, all the experimental efforts to isolate the imine intermediate **2** have so far proved unsuccessful, likely due to the instability of such intermediate in solution.^38^ Given this limitation, molecular modeling techniques are an indispensable solution to investigate the mechanistic details of the biotransformation of **2**. Due to the similarity of the chemical skeleton of imine **2** and oxime **1**, it is very likely that the imine **2** binds the same way as the oxime **1** in . Also, our previous MD simulations indicated that both **1** and **2** have similar binding poses.^38^ Additionally, the docked pose of **2** is also very similar to our modeled complex of **2** in the active site of XenA, which has been modeled by using the binding pose of **1** as a template. The structural superimposition of the docked pose and our QM/MM optimized binding pose of **2** is shown in Figure S13a. We have considered the neutral form of **2** instead of a positively charged iminium ion based on the calculated p*K*_a_ value for the imine (0.0), rendering it very unlikely that **2** is protonated at neutral pH. The geometric features of the QM/MM optimized complex of XenA with **2** are similar to the one with **1** (Table S2). For example, the distance between the N1 atom of **2** and the flavin’s N5 atom is within 3.5 Å, and the N10–N5–N1 angle is close to 90°. Additionally, the O3 atom of **2** has two hydrogen bonds with H178 and H181 (Figure S13b), the latter being closer. Overall, the QM/MM optimized binding pose of substrate **2** has its N1 atom in a very familiar position for receiving a hydride ion. The polar interactions between the substrate **2** and the active site residues have been summarized in Figure S13b.

### Reaction Mechanism

#### Hydride Transfer Occurs First, Followed by Proton Transfer

Imine **2** is proposed to receive a hydride from FMNH^–^, and a conserved tyrosine residue (Y183) or a nearby water molecule is identified as a potential proton source. Overall, the proposed mechanism yields an amine. However, it is not known whether the proton and the hydride are added together or in distinct steps. 2D potential energy surfaces were generated with the hydride and proton transfer reaction coordinates ( and ) varied simultaneously to determine the order of events. We have found that like substrate **1**, substrate **2** also undergoes a two-step reaction, accepting the hydride first, followed by proton transfer (Figure S14). This mechanism aligns with other reported catalytic reactions of OYE, where the flavin cofactor reduces the C=C via a hydride transfer followed by a proton from the solvent or the enzyme itself.^28,31,52,57^

### Hydride Transfer Step

#### Structural Analysis of Reaction Coordinates

**Figure 4:**
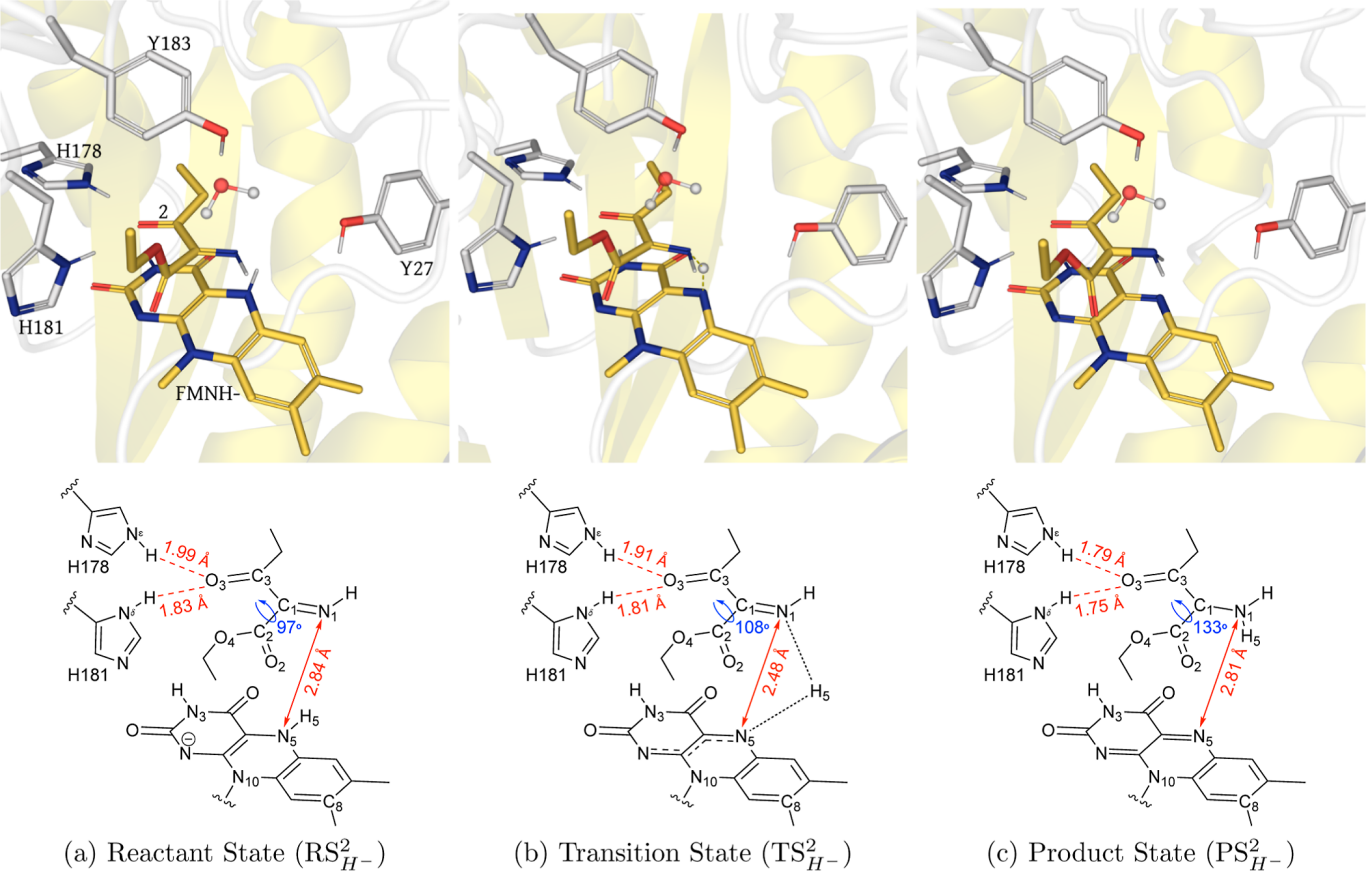
(Top) QM/MM (UB3LYP-6-31G*/OPLS2005) geometry optimized structures of the active site of XenA in the (), (), and () configurations with **2** during the hydride transfer. The substrate **2** and LuF are shown in yellow sticks, side chains of active site residues are shown in light gray sticks, and water is displayed in the ball and stick representation. In the () structure, the transient H5 atom is also depicted as a gray ball. (Below) Schematic of each configuration showing key distances (red) and dihedrals (blue).

The substrate **2** also has all of the similar notable features of the typical OYE substrates, as we discussed before in the case of substrate **1**. The optimized molecular structures corresponding to stationary points along the lowest energy reaction coordinate for the hydride transfer are displayed in Figure 4, mentioning the key geometrical parameters. Here again, the hydride transfer modulates geometrical changes similar to those in the case of substrate **1** (Figure S15). Notably, the distance between the H5 and acceptor N1 decreases from 2.10 Å in  to 1.29 Å in , and then to 1.02 Å in . Whereas the distance between the donor N5 and acceptor N1 decreases from 2.84 Å in  to 2.48 Å in , and then increases to 2.81 Å in . The isoalloxazine ring also gets flatter as the H5 atom approaches the N1 atom (Figure S15c). Interestingly, a marginal difference between the NδH–O3 and NεH–O3 distances also exists here. Another similar influence of the delocalization can be seen over the dihedral angle (C3–C1–C2–O2), which increases to 108° in  from 97° in , to facilitate a better orbital overlap between π(C1=N1) and the π*(C2=O2). In , the aforementioned Bürgi-Dunitz angle for the hydride approaching the C1=N1 bond is 111.2°, which is also similar to the value found in . The calculated Δ*E*_a_^‡^ and Δ*G*^‡^ for the hydride transfer to **2** are 12.46 and 6.34 kcal/mol (Figure S16), respectively. Notably, the calculated energy barriers for the hydride transfer to **2** are lower than the same calculated for **1**. In fact, in a couple of our 5 ps QM/MM production runs for substrate **2**, we observed that the hydride was transferred to N1 without any external force or bias, whereas this spontaneous hydride transfer was not seen during any of the three 5 ps QM/MM production runs for **1**. The computed enthalpy of this hydride transfer reaction is −0.15 kcal/mol.

### NBO Analysis of Reaction Coordinates

Like substrate **1**, the vacant π*(C1=N1) NBO is the leading target orbital for the delocalization of the electrons from the donor σ(N5–H5) NBO (Figure S17). At the same time, the native electron density of π(C1=N1) NBO is delocalized to the neighboring vacant orbitals, primarily to the π*(C3=O3) NBO. The contrasting variability across intramolecular bonds (C1–N1, C1–C3, and C3–O3) along with the changes in net atomic charge on atoms (C1, C3, O2, O3, and N1) articulate the effect of delocalization (Figure S18). Interestingly, the N1 atom is more negatively charged than its counterpart in **1**. We argue that substrate **2** lacks the electronegative hydroxyl oxygen atom attached to N1, which could draw considerable electron density from the N1 atom. While the hydride approached the N1 atom, the C1 atom started looking for vacant orbitals to share a pair of electrons; surprisingly, instead of Y183, a water molecule approached the C1 atom, directing one of its empty σ*(Hw-Ow) toward the electron-rich lone-pair orbital of C1. The corresponding *E*^2^ value between the LP(C1)-σ*(Hw-Ow) is shown in Figure S19.

#### Proton Transfer Step

**Figure 5:**
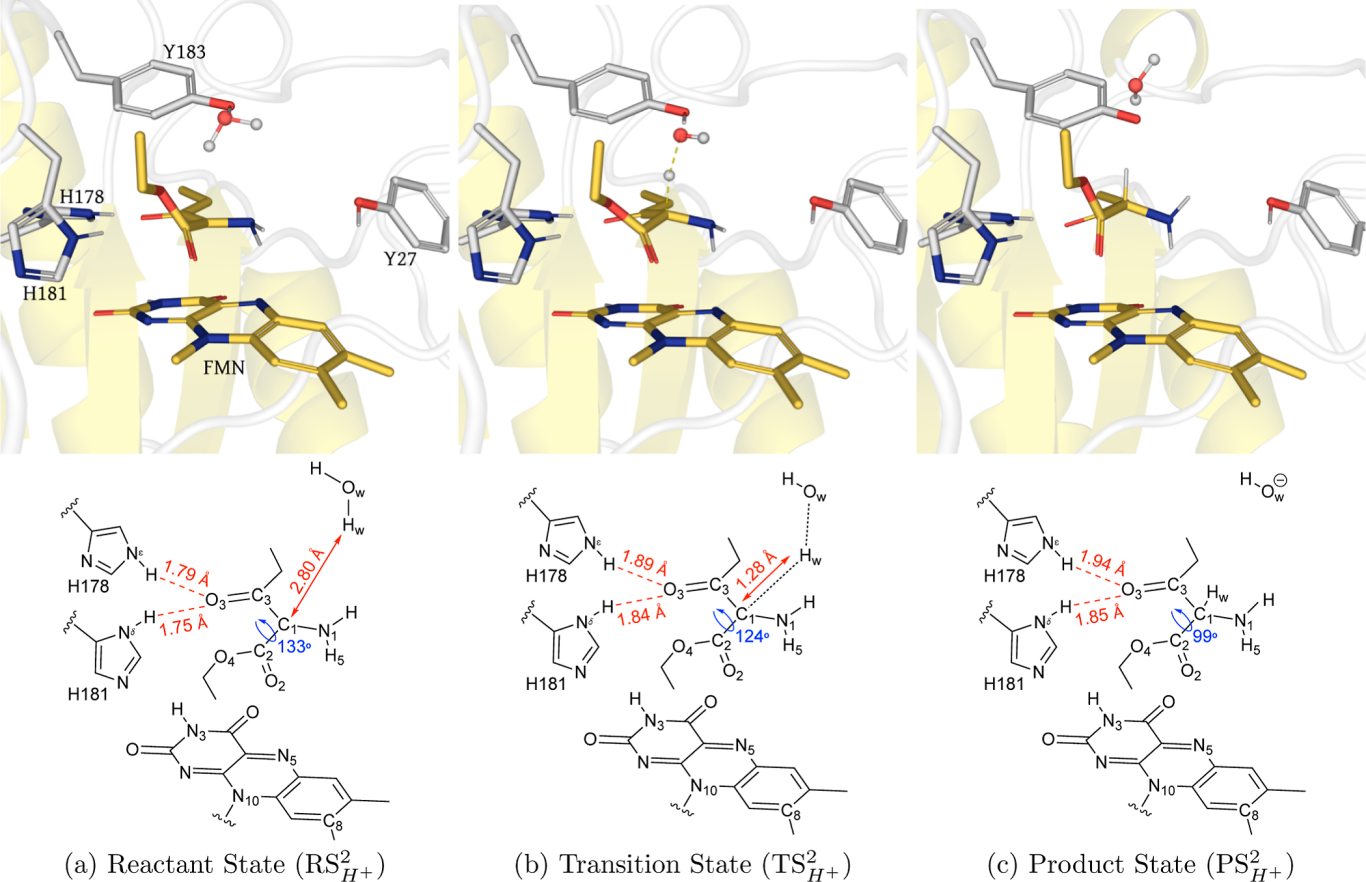
(Top) QM/MM (UB3LYP-6-31G*/OPLS2005) geometry optimized structures of the active site of XenA in the (), (), and () configurations with **2** during the proton transfer. The substrate **2** and LuF are shown in yellow sticks, side chains of active site residues are shown in light gray sticks, and water is displayed in the ball and stick representation. In the () structure, the transient Hw atom is also depicted as a gray ball. (Bottom) Schematic of each configuration showing key distances (red) and dihedrals (blue).

We have used the product state  from the hydride transfer step as the reactant state  for the investigation of the proton transfer step. Because of the hydride transfer, the N1–C1 bond becomes more polarized than in . From here, C1 could fetch a proton from nearby water molecules or Y183; we have considered both possibilities and found that energetically, a direct proton transfer from Y183 is marginally more favorable than an indirect proton transfer from the Y183 via solvent (Figure S20). Lonsdale et al. also drew a similar conclusion when comparing proton transfer from the Y169 (counterpart of Y183 in YqjM) or water to Cα carbon in α–β unsaturated ketones.^52^ Optimized molecular structures corresponding to stationary points along the lowest energy reaction coordinate for the direct proton transfer from Y183 are shown in (Figure S21); here, we consider water as a proton donor for the general case. The optimized molecular structures corresponding to stationary points along the lowest energy reaction coordinate for water-mediated proton transfer are shown in Figure 5. As the proton distances itself from its source and approaches the C1 atom, the hydrogen bonding distances of O3 with H178 and H181 become longer and longer due to the electron relocalization. Similar to the proton transfer reaction of **1**, the water molecule removes a proton from Y183 while losing its native proton to C1. Interestingly, in , the approaching proton forms an obtuse angle with the C1=N1 bond, similar to the one the approaching hydride had in , but in the opposite direction. The dynamic evolution of geometrical parameters discussed here is presented in (Figure S22). The calculated Δ*E*_a_^‡^ and Δ*G*^‡^ for the proton transfer from the Y183 are 12.62 and 13.65 kcal/mol, respectively. The computed enthalpy for the same is 0.35 kcal/mol. The calculations indicate that the proton transfer reaction is endergonic (Figure S20). This is very likely due to charge separation where the side chain of Y183 became negatively charged and water molecules could protonate the Y183 in a subsequent step. Similar observations were made in previous QM/MM studies on OYEs.^52,58^ Notably, the overall energy barrier for the reduction of the C=N double bond of **2** is lower than the one reported for the reduction of C=C (17.0 kcal/mol) by the same class of OYE.^52,59^ However, the hydride transfer was the rate-limiting step for the reduction of C=C,^52^ whereas in **2**, the proton transfer is slower than the hydride transfer.

### NBO Analysis of Reaction Coordinates

The accumulated electron density on the C1 atom of **2** due to the hydride transfer reaction pushes the C1 atom to act like a Lewis base. A nearby solvent molecule is arguably the most approachable Lewis acid. Consequently, after losing an H^+^ to the C1 atom, the Ow-H atom becomes a Lewis base itself and draws a proton from another Lewis acid, namely Y183. Eventually, Y183 indirectly donates a proton via a water molecule. This can be seen in *E*^2^ value plot between the donor–acceptor NBOs, where at first, the donor LP(C1) delocalized its electron density on acceptor σ*(Hw-Ow) to draw a proton (Hw^+^), followed by the similar interaction of donor LP(Ow) with acceptor σ*(Y183–O–H) (Figure S23). The resonance stabilized negatively charged side chain of the Y183 started approaching its surroundings, and in favor, the nearby water molecules have shown considerable polar interactions. At the end of this reaction, the substrate’s atomic charges and bond lengths are approaching their original values at the beginning of the reaction in  (Figure S24). Except for the C1–N1 bond, which is now a single bond after losing its π electrons to empty 1s orbital of added *H*^+^, in contrast, the carbonyl (C3=O3) bond became shorter and less polarized.

## Conclusions

We employed a combination of advanced QM/MM simulations and NBO analyses to investigate the molecular details of the two-step conversion of oximes to amines catalyzed by ene-reductases. While our study specifically examined the reductions of **1** and **2** using XenA, our results should have broader implications for understanding similar OYE-catalyzed biotransformations. The modeled binding modes of oxime **1** and imine **2** are structurally very similar to those observed for other OYE substrates, such as α, β-unsaturated ketones, and lend themselves well to the oxidation of the FMNH^–^ cofactor. We found that the hydride transfer to the nitrogen atom in **1** is followed by a spontaneous water-mediated proton transfer from Y183 to the oxime oxygen, leading to the dehydration of the substrate. This pattern is repeated in the subsequent reduction of imine **2**, however, involving an energetically more favorable direct protonation by Y183 rather than by solvent molecules. Hydride transfer was found to be the rate-limiting step in the conversion of oxime **1** to corresponding imine intermediate **2**, while proton transfer was rate-limiting for imine reduction. Our simulation results and calculated energy barriers confirm the previously proposed catalytic mechanism and agree well with experimental mutagenesis and enzyme kinetics data.^38^ In particular, the NBO analyses reveal the crucial role of electron delocalization, which is enhanced by active site residues as well as by substituents attached to the C=N bond of the substrates. This delocalization facilitates the unconventional hydride transfer to the C=N bond by spreading the incoming electron pair across the substrate rather than confining it to a specific atom or bond. Our study also stresses the significance of analyzing the dynamics of the active site residues when investigating enzymatic reactions. This was particularly evident from the dynamic changes in the interaction strengths between two active site histidine residues, H178 and H181, and the keto group of the substrate during hydride transfer. As the reaction approached the transition state, the substrates also underwent conformational changes that aligned the electron-withdrawing groups to the C=N bond, thereby optimizing electron delocalization. These dynamic changes along a reaction path can arguably be studied in detail only with the help of computer simulations. They offer deep insights into catalytic mechanisms and provide valuable input for enzyme and substrate engineering efforts.

## Supporting Information Available

Description of the employed methodology, as well as supplementary schemes, figures, and tables; and QM/MM geometry optimized structures for each reaction coordinate, the input files for the QM/MM simulations, and a detailed tutorial explaining the methodology of this work are publicly available on GitHub (https://github.com/hopanoid/Enzyme-Reaction-Dynamics-Tutorial) (PDF)
