# Supplementary material for: Deciphering the Unconventional Reduction of C=N Bonds by Old Yellow Enzymes Using QM/MM
Source: ACS Catal. 2024 Jan 10;14(3):1257–66. doi: 10.1021/acscatal.3c04362 (PMC10845114; doi:10.1021/acscatal.3c04362)
Supplement: Supplementary file 1 — cs3c04362_si_001.pdf [file cs3c04362_si_001.pdf]

Supplementary Information

Deciphering the Unconventional Reduction of C=N Bonds by Old Yellow Enzymes using QM/MM

Amit Singh Sahrawat,[†] Nakia Polidori,[†] Wolfgang Kroutil,[‡,¶,§] and Karl Gruber[*,†,¶,§]

†*Institute of Molecular Biosciences, University of Graz, Graz, 8010, Austria*

‡*Institute of Chemistry, University of Graz, Graz, 8010, Austria*

¶*Field of Excellence BioHealth, University of Graz, Graz, 8010, Austria*

§*BioTechMed-Graz, Graz, 8010, Austria*

E-mail: karl.gruber@uni-graz.at

Supplementary Figures & Tables

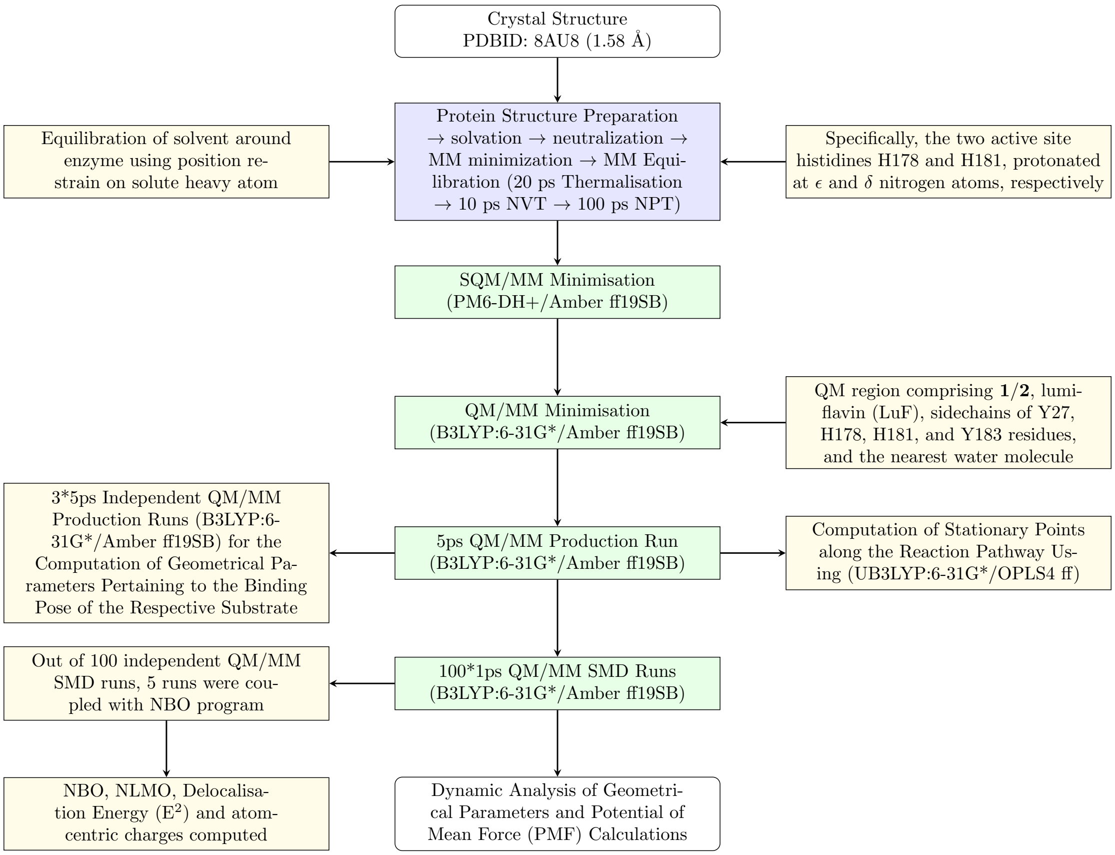

Scheme S1: Summary of Methodology.

Table S1: Summary of key geometric parameters of the QM /MM optimised binding pose of oxime **1**. Data were obtained from 3 independent 5 ps QM /MM SMD simulations.

Geometric Parameter[Unit]	Avg±SD
d(NδH-O3)[Å]	2.30±0.50
d(NεH-O3)[Å]	3.57±1.15
d(N5-N1)[Å]	3.32±0.43
$\angle$(N10-N5-N1)[°]	79.66±18.39
$\angle$(C3-C1-C2-O2)[°]	115.75±25.86

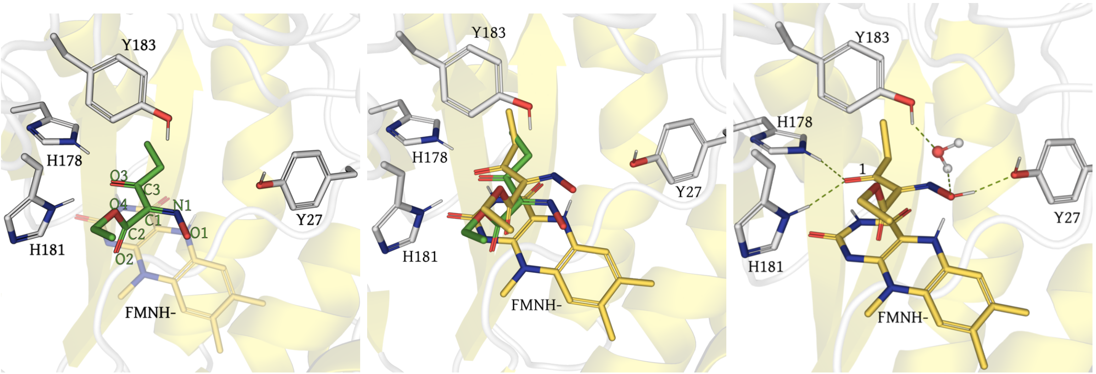

(a) (b) (c)

Figure S1: Active site of XenA in complex with **1**. (a) Crystal structure of the complex (PDB entry: 8AU8) with the key atoms of **1** labelled in green. (b) Structural alignment of the binding poses of **1** as observed in the crystal structure (stick representation with green carbons) and obtained by QM /MM (UB3LYP-6-31G*/OPLS2005) optimisation (yellow carbons). (c) Polar interactions between the substrate and the active site residues have been shown in green dashed lines. The active site residues are shown in a stick representation with grey-coloured carbons, while the lumiflavin (LuF) carbons are coloured yellow. The alignment was performed using the pair-fitting method available in Pymol,[1] where the corresponding pairs of heavy atoms were selected from the side chains of the active site residues and LuF. For clarity, the ribitylphosphate tail of the flavin cofactor and nonpolar hydrogen atoms of the enzyme are hidden.

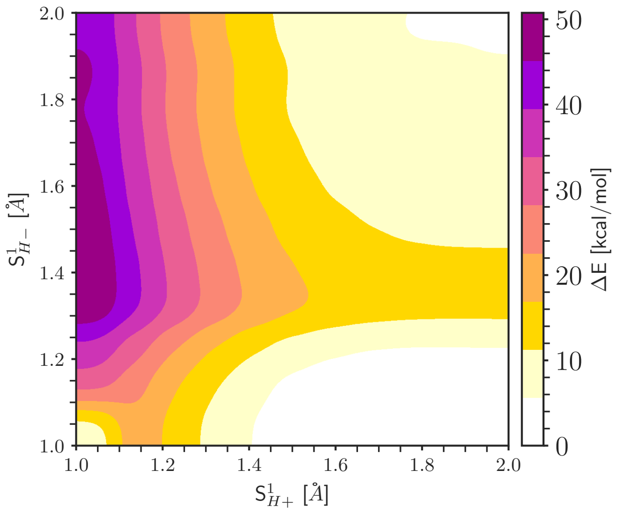

Figure S2: 2D potential energy surface for the conversion of **1** to **2** by XenA from *Pseudomonas putida* (calculated at the UB3LYP-6-31G*/OPLS2005 level). We performed a scan along the reaction coordinate of the hydride transfer (S^1_{H-}). Then, each optimised structure was used as a starting point for the proton transfer reaction coordinate (S^1_{H+}) with the hydride transfer coordinate fixed. The reaction coordinates were varied in 0.1 Å steps. The details of S^1_{H-} and S^1_{H+} are shown in Supplementary Figure S27a. The figure was drawn with the imshow function of Matplotlib using the Gaussian interpolation method.

Table S2: Summary of key geometric parameters of the QM/MM optimised binding pose of imine **2**. Data were obtained from 3 independent 5 ps QM/MM (B3LYP-6-31G*/amber19ffsb) SMD simulations.

Geometrical Parameter[Unit]	Avg±SD
d(NδH-O3)[Å]	2.05±0.21
d(NεH-O3)[Å]	2.75±0.45
d(N5-N1)[Å]	3.04±0.31
∠(N10-N5-N1)[°]	85.36±11.37
∠(C3-C1-C2-O2)[°]	130.20±25.89

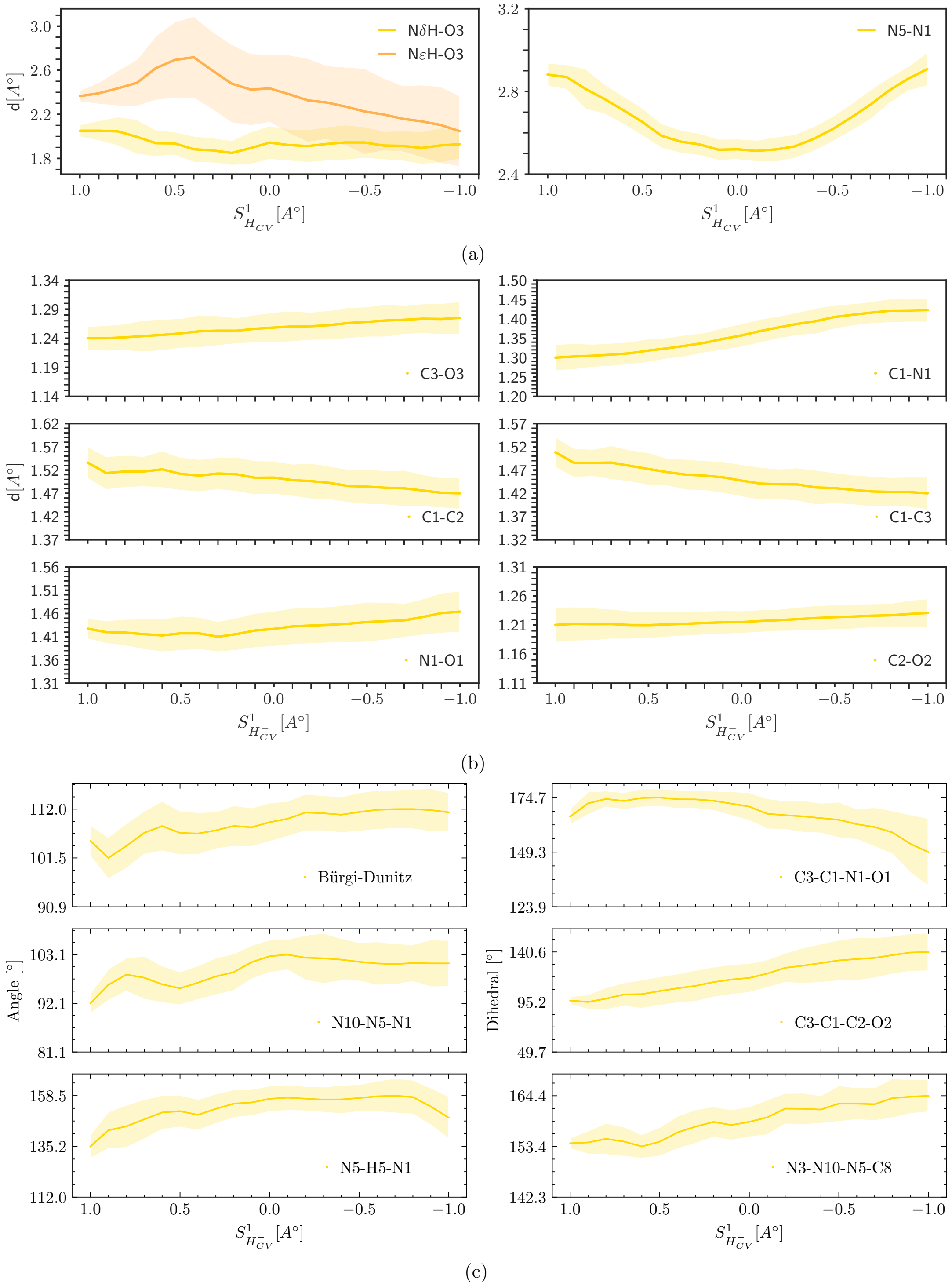

Figure S3: Dynamics of geometric changes across the reaction coordinates during the hydride transfer step for **1**. (a) Intermolecular distances between **1** and XenA, (b) intramolecular distances within **1**, and (c) selected angles and dihedrals between **1** and XenA. The plots were generated using data from 100 independent 1 ps QM /MM (B3LYP-6-31G*/amber19ffsb) SMD simulations. The standard deviations are shown as semi-transparent bands.

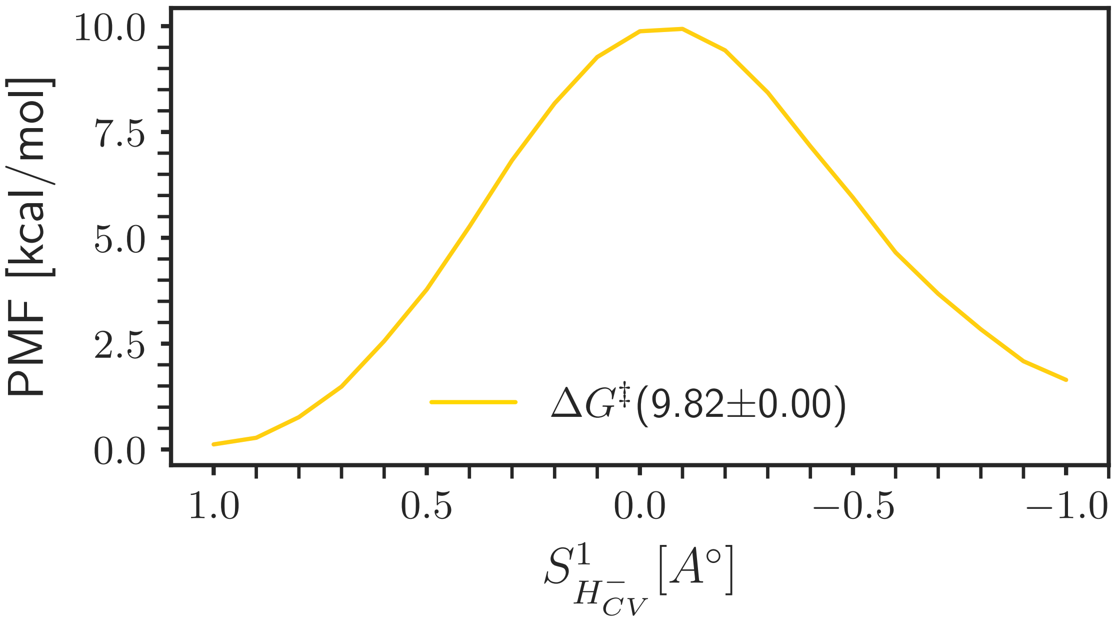

Figure S4: Potential of mean force (PMF) across the reaction coordinates during the hydride transfer step for **1**. The data were derived from the 100 independent 1 ps QM/MM (B3LYP-6-31G*/amber19ffsb) SMD simulations. The mean square errors are shown as vertical bars.

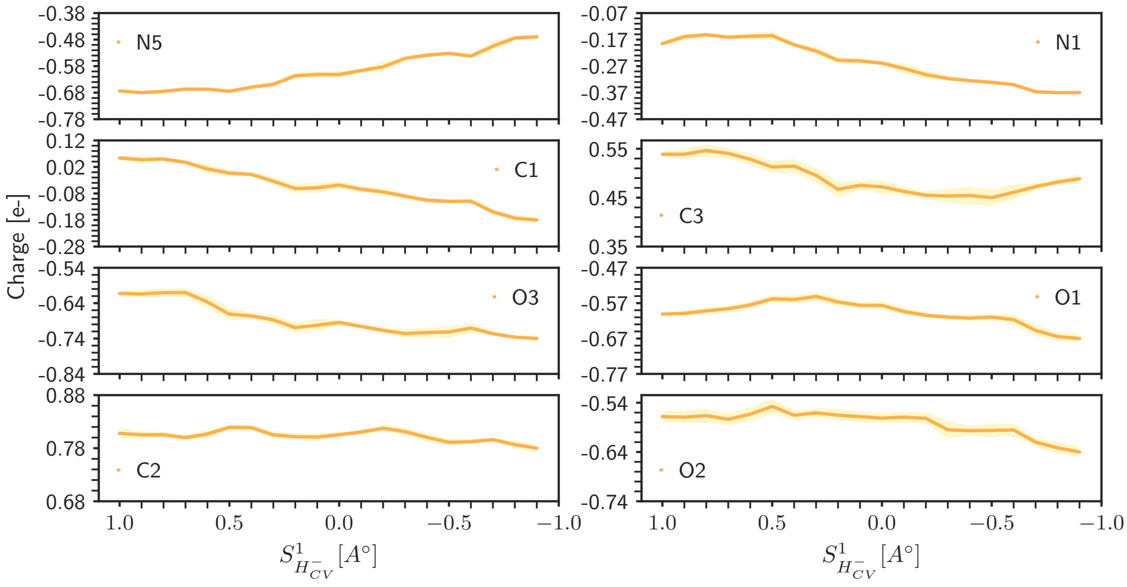

Figure S5: Dynamics of atom-centred charges versus reaction coordinates during the hydride transfer step for **1**. The plots were generated using data obtained with the NBO program from 5 independent 1 ps QM/MM (B3LYP-6-31G*/amber19ffsb) SMD simulations. The standard deviations are shown as semi-transparent bands.

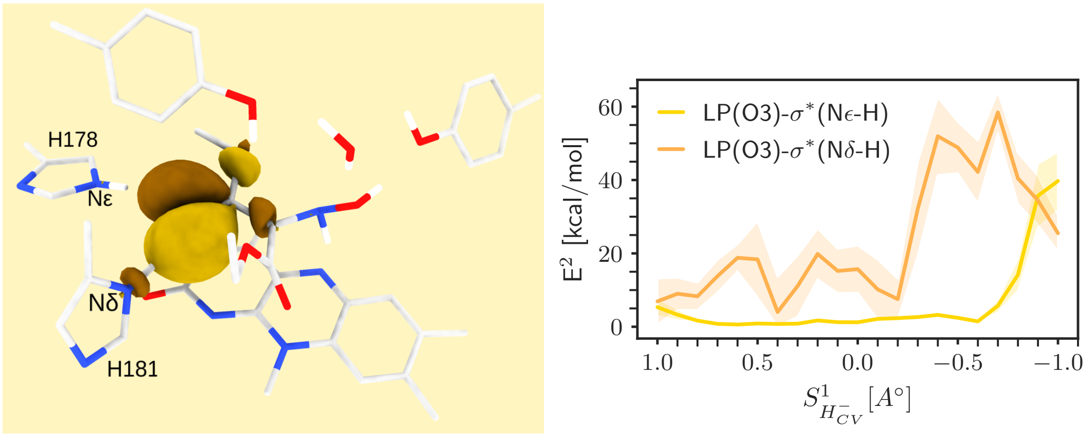

(a) (b)

Figure S6: Role of H178 and H181 in stabilising the accumulated electron density on **1** during the hydride transfer reaction. (a) A snapshot from a QM/MM (B3LYP-6-31G*/amber19ffsb) SMD trajectory showing the proximity of H181 to the lone pair of O3. (b) The delocalisation energies $E^{(2)}$ are plotted together with the standard deviations shown as semi-transparent bands.

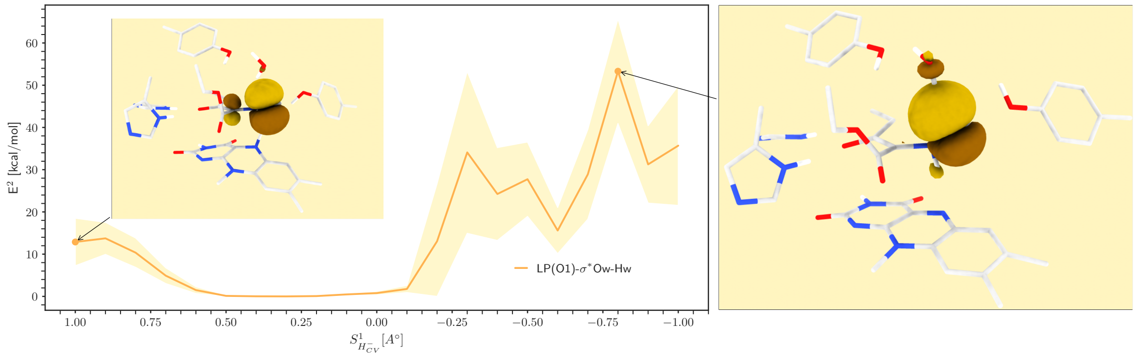

Figure S7: NBO analysis across the reaction coordinates during the hydride transfer step for **1**. Plot of the delocalisation energy (E^2) between the donor LP(O1) and the acceptor σ^*(Hw-Ow) NBOs. The standard deviations are shown as semi-transparent bands, while the two insets show the extent to which the LP(O1) donor NBO is perturbed due to delocalisation.

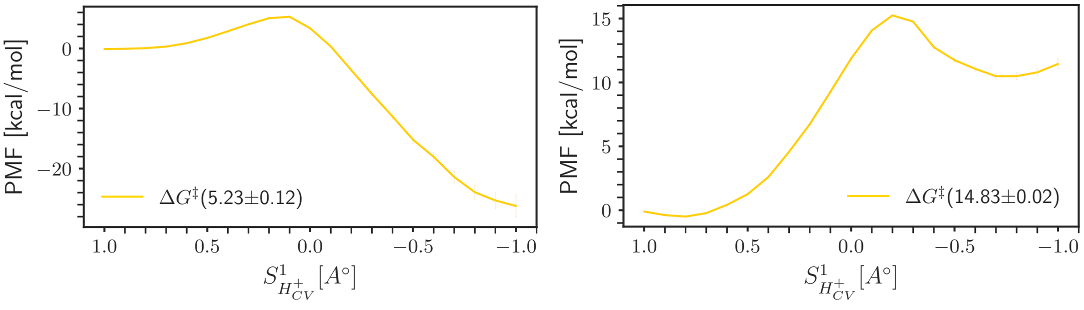

(a) (b)

Figure S8: Potential of mean force (PMF) across the reaction coordinates during the proton transfer step for **1** via water (a), or via Y27 (b). Data were derived from 100 independent 1 ps QM/MM (B3LYP-6-31G*/amber19ffsb) SMD simulations. The mean square errors are shown as vertical bars.

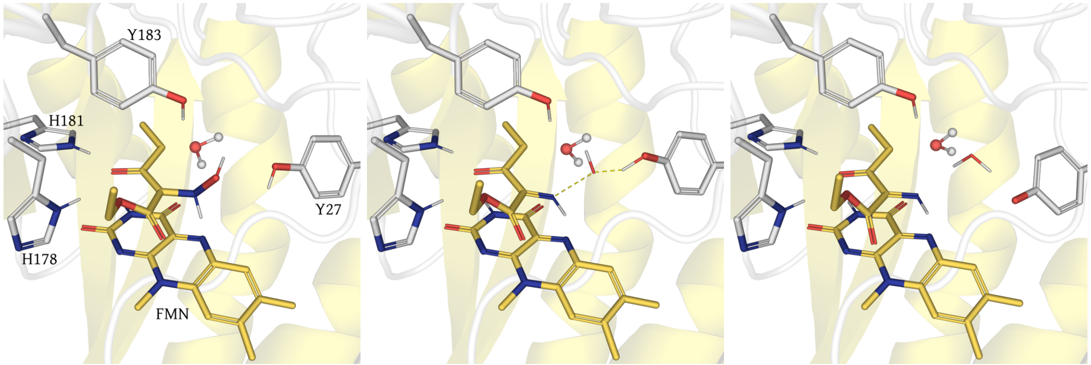

(a) Reactant State (RS^1_{H+}) (b) Transition State (TS^1_{H+}) (c) Product State (PS^1_{H+})

Figure S9: QM/MM (UB3LYP-6-31G*/OPLS2005) geometry optimised structures of the active site of XenA in the (RS^1_{H+}), (TS^1_{H+}) and (PS^1_{H+}) configurations with **1**. The configurations shown are the optimised molecular structures corresponding to stationary points along the lowest energy reaction coordinate for the direct proton transfer from Y27.

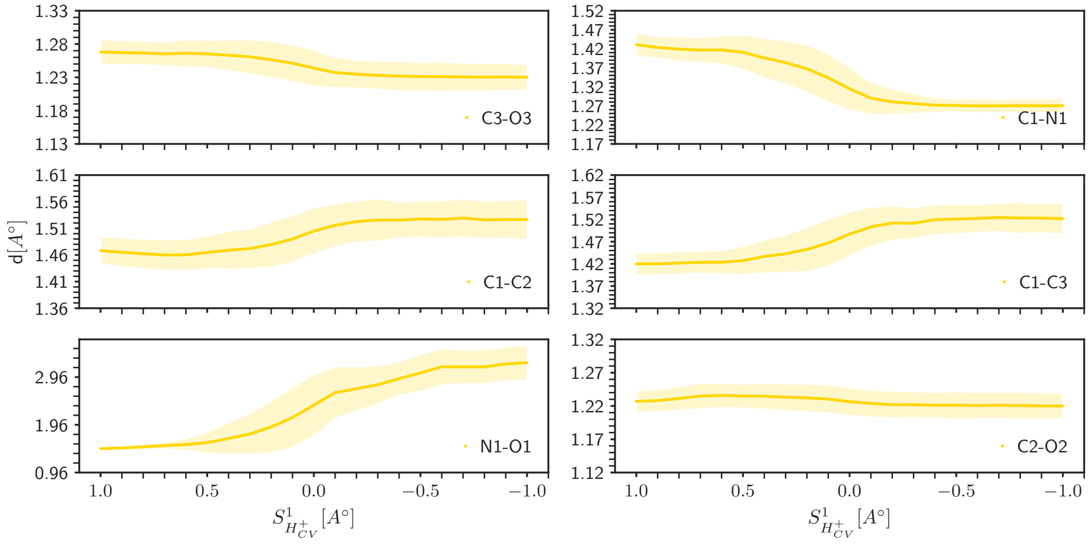

(a)

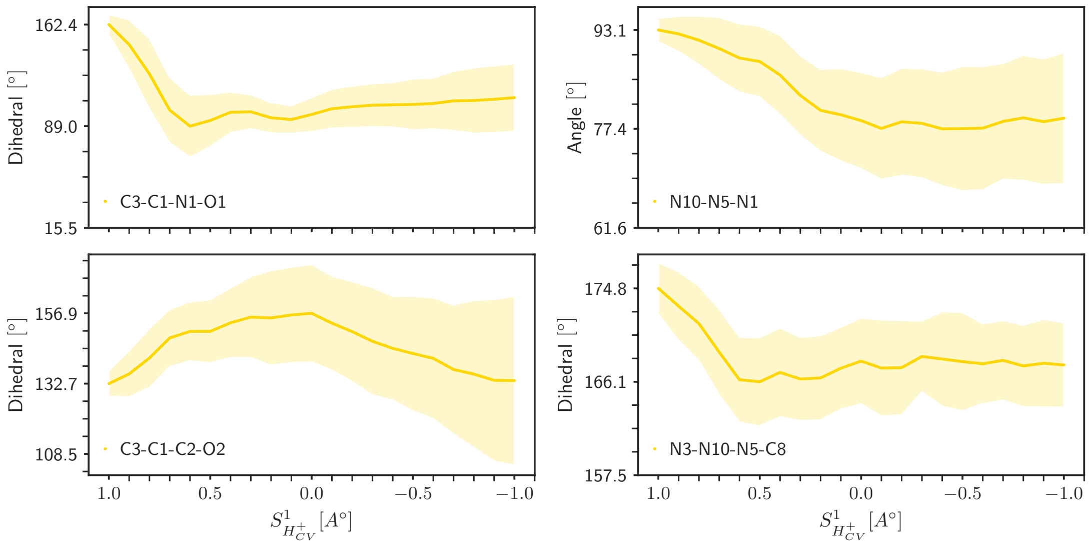

(b)

Figure S10: Dynamics of geometric changes across the reaction coordinates during the proton transfer step for **1**. (a) Intramolecular distances within **1**, and (b) selected angles and dihedrals between **1** and XenA. The plots were generated using data from 100 independent 1 ps QM/MM (B3LYP-6-31G*/amber19ffsb) SMD simulations. The standard deviations are shown as semi-transparent bands.

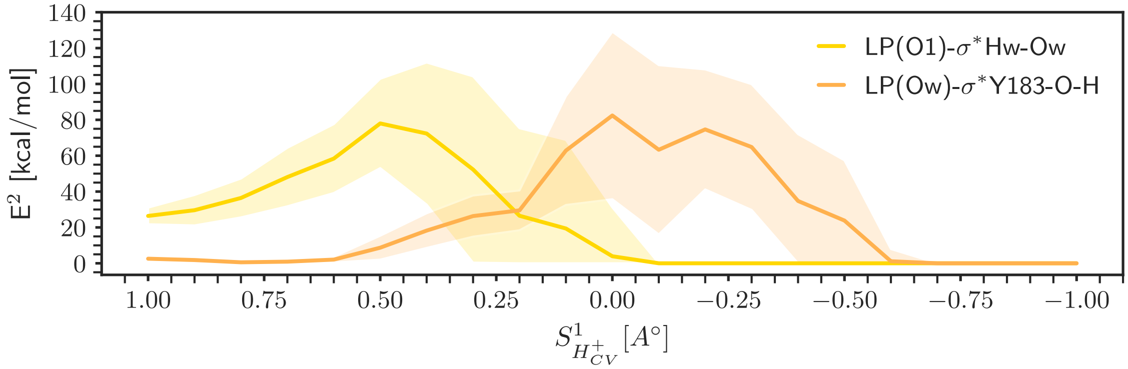

Figure S11: NBO analysis across the reaction coordinates during the proton transfer step for **1**. Delocalization energies (E^2) between the respective pairs of donor-acceptor NBOs are plotted together with the respective standard deviations shown as semi-transparent bands.

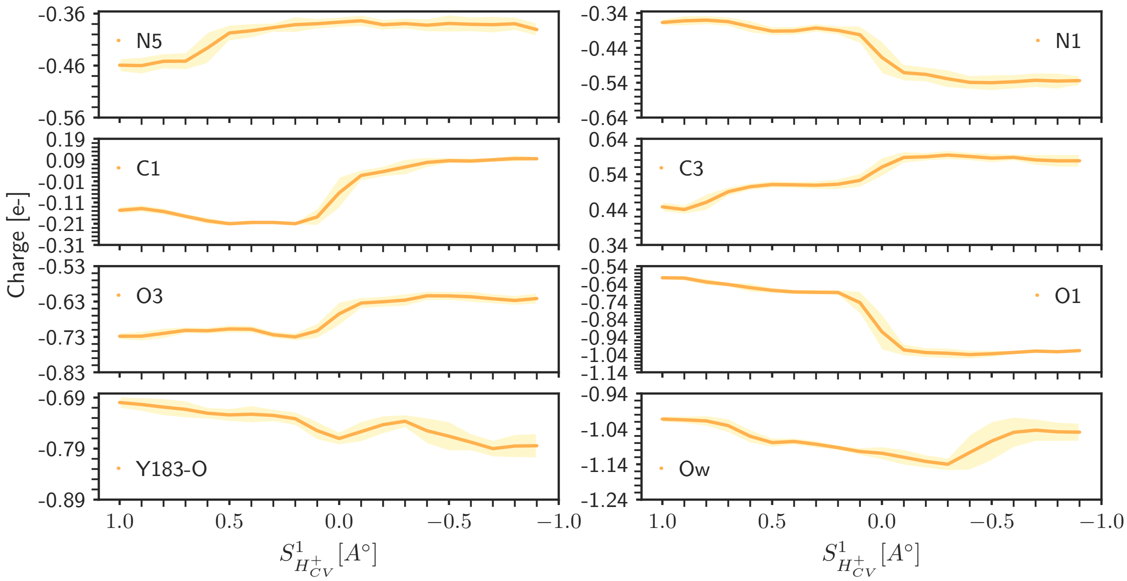

Figure S12: Dynamics of atom-centred charges across the reaction coordinates during the proton transfer step for **1**. The plots were generated using data obtained with the NBO program from 5 independent 1 ps QM/MM (B3LYP-6-31G*/amber19ffsb) SMD simulations. The standard deviations are shown as semi-transparent bands.

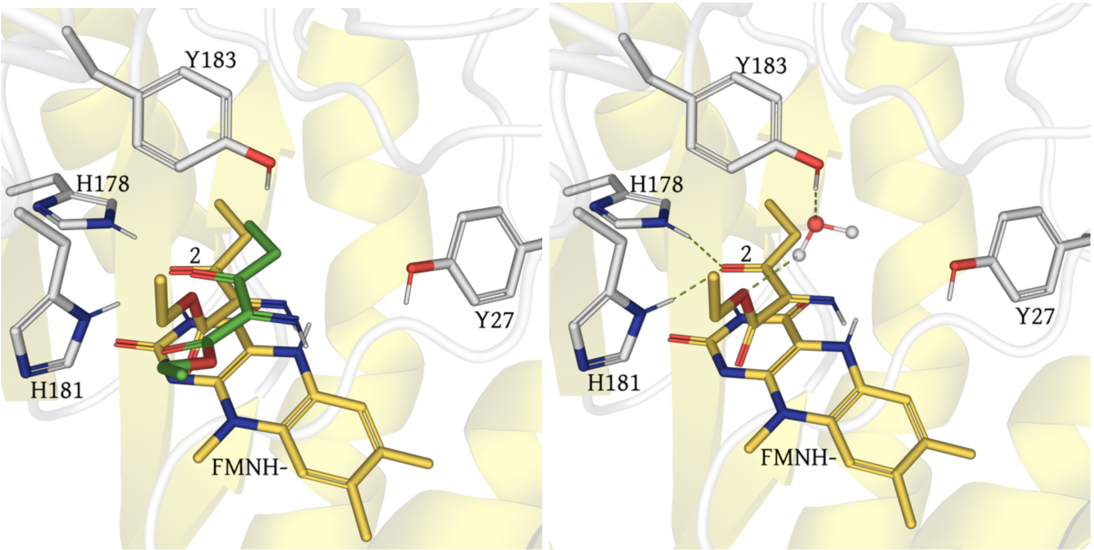

(a) (b)

Figure S13: Active site of XenA in complex with **2**. (a) Structural alignment of the docking pose of **2** (stick representation with green carbons) and obtained by QM /MM (UB3LYP-6-31G*/OPLS2005) optimisation (yellow carbons). (b) Polar interactions between the substrate and the active site residues have been shown in green dashed lines. The active site residues are shown in a stick representation with grey-coloured carbons, while the lumiflavin (LuF) carbons are coloured yellow. The alignment was performed using the pair-fitting method available in Pymol,[1] where the corresponding pairs of heavy atoms were selected from the side chains of the active site residues and LuF. For clarity, the ribitylphosphate tail of the flavin cofactor and nonpolar hydrogen atoms of the enzyme are hidden.

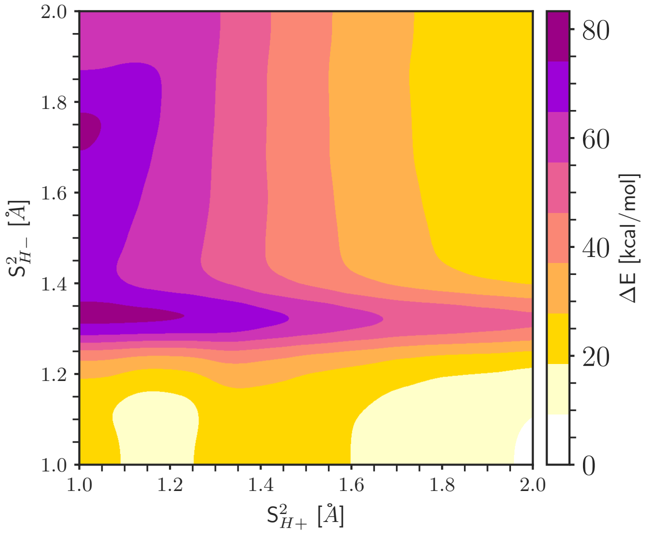

Figure S14: 2D potential energy surface for the conversion of **2** to **3** by XenA from *Pseudomonas putida* (calculated at the UB3LYP-6-31G*/OPLS2005 level). We performed a scan along the hydride transfer reaction coordinate (S^2_{H-}). Then, each optimised structure was used as a starting point for the proton transfer reaction coordinate (S^2_{H+}) with the hydride transfer coordinate fixed. The reaction coordinates were varied in 0.1 Å steps. The details of S^2_{H-} and S^2_{H+} are mentioned in supplementary figure S27b. The figure was drawn with the imshow function of Matplotlib using the Gaussian interpolation method.

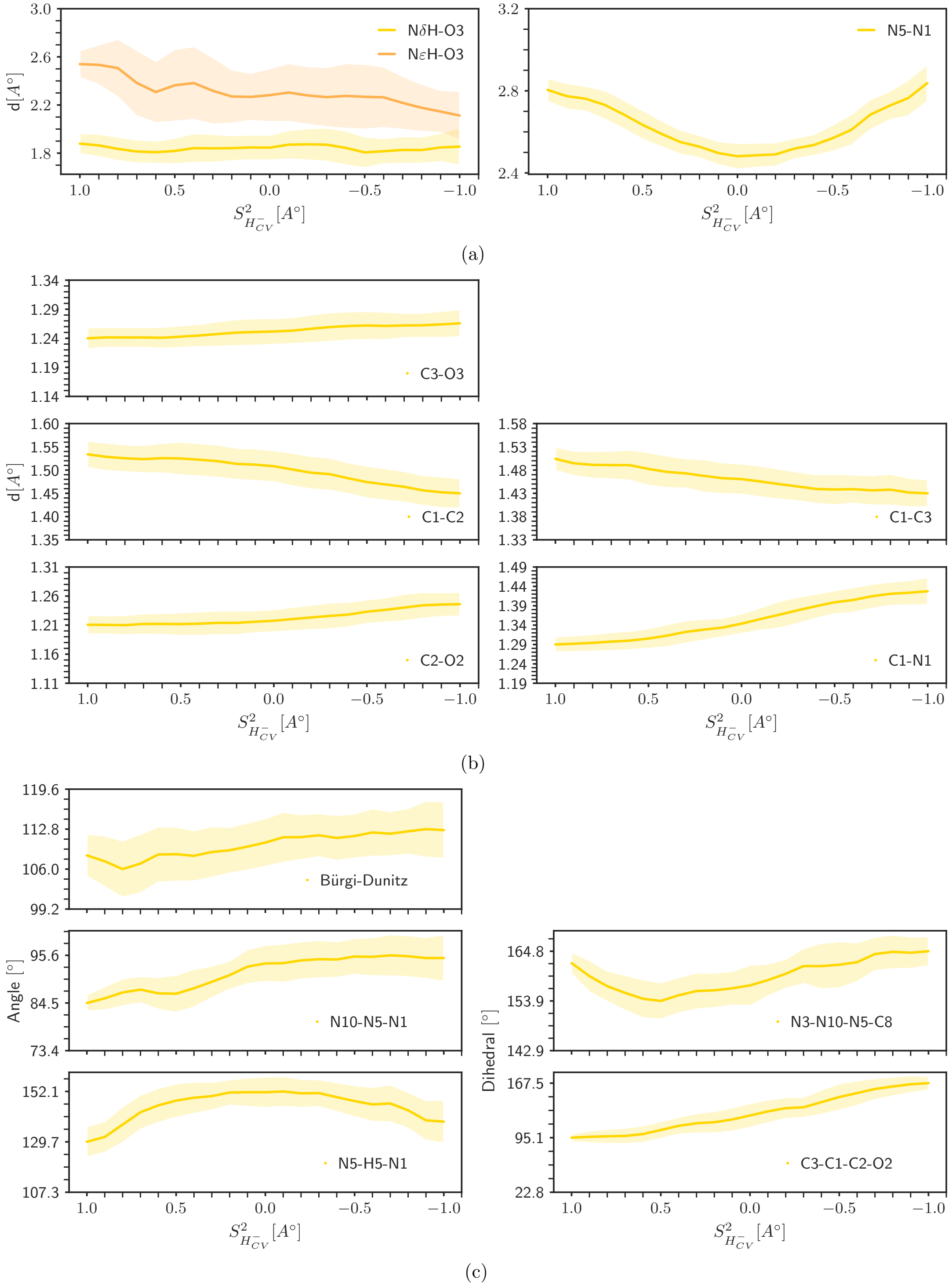

Figure S15: Dynamics of geometric changes across the reaction coordinates during the hydride transfer step for **2**. (a) Intermolecular distances between **2** and XenA, (b) Intramolecular distances within **2**, and (c) selected and dihedrals between **2** and XenA. The plots were generated using data obtained from 100 independent 1 ps QM/MM (B3LYP-6-31G*/amber19ffsb) SMD simulations. The standard deviations are shown as semi-transparent bands.

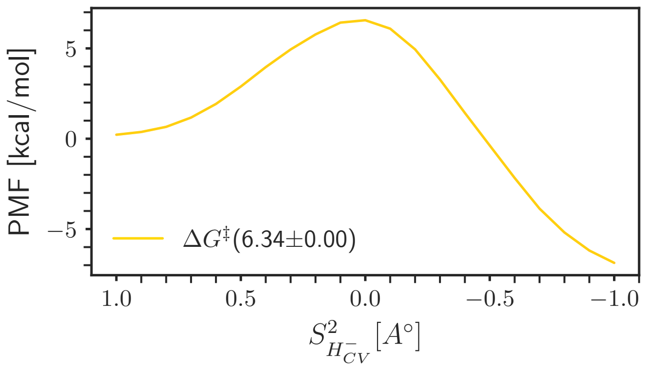

Figure S16: Potential of mean force (PMF) across the reaction coordinates during the hydride transfer step for **2**. The data were derived from the 100 independent 1 ps QM /MM SMD simulations. The mean square errors are shown as vertical bars.

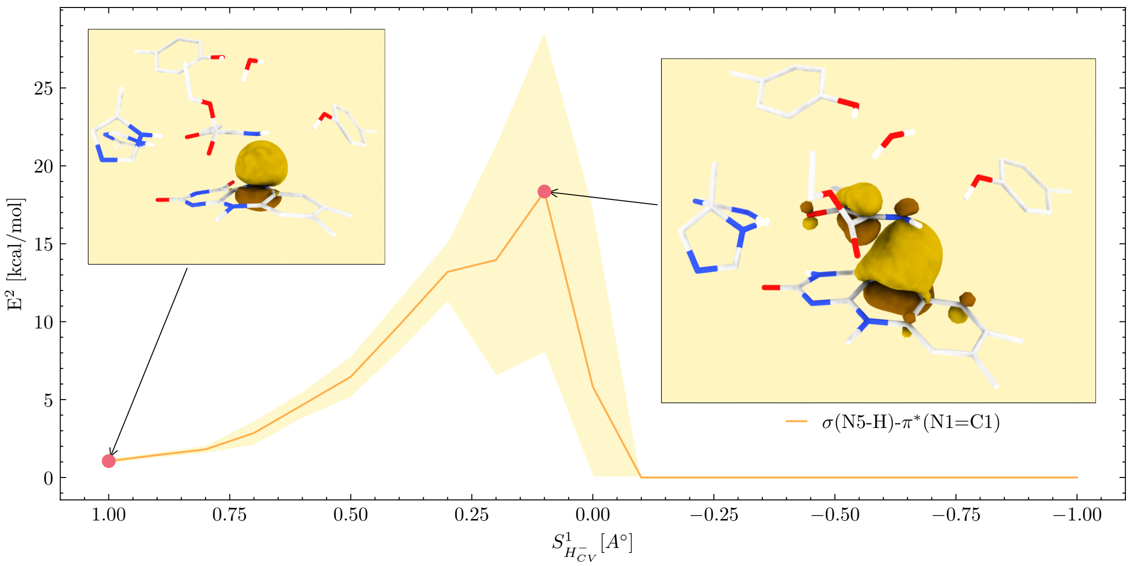

Figure S17: NBO analysis across the reaction coordinates during the hydride transfer step for **2**. Plot of the delocalization energy (E^2) between the donor σ(N5-H5) and acceptor π^*(C1=N1) NBOs for **2**. The standard deviations are shown as semi-transparent bands, while the two insets show the extent to which the σ(N5-H5) donor NBO is perturbed due to delocalisation.

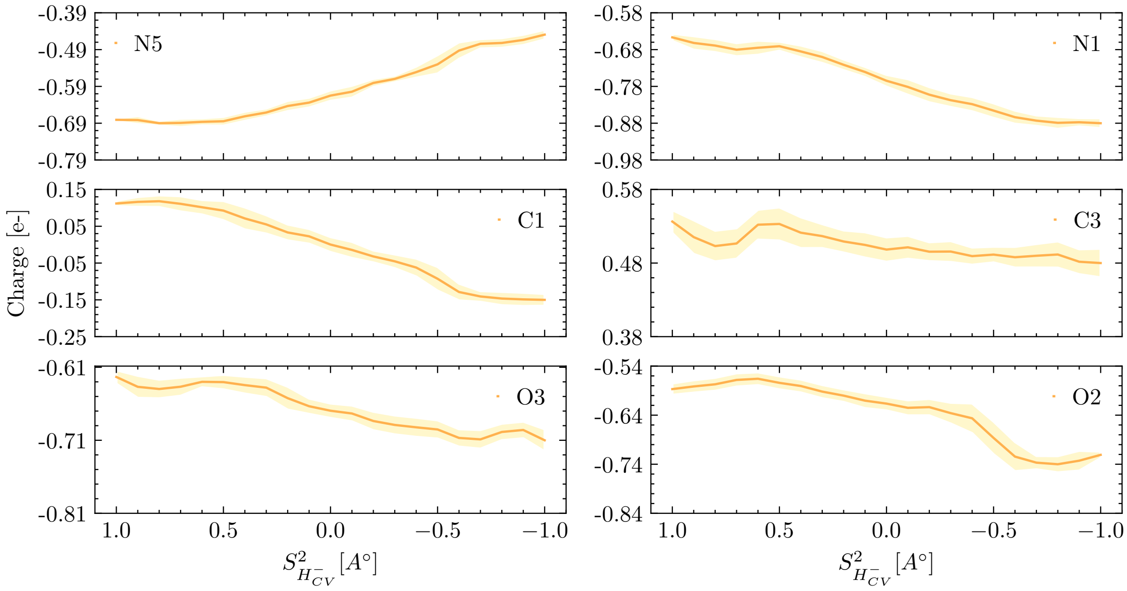

Figure S18: Dynamics of atom-centred charges across the reaction coordinates during the hydride transfer step for **2**. The plots were generated using data obtained with the NBO program from 5 independent 1 ps QM/MM (B3LYP-6-31G*/amber19ffsb) SMD simulations. The standard deviations are shown as semi-transparent bands.

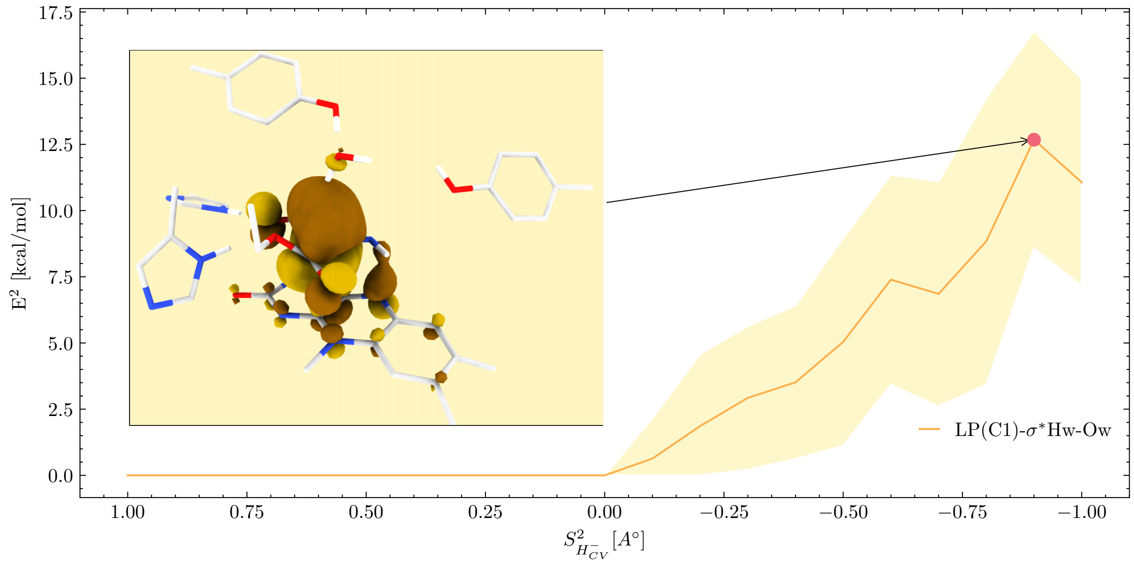

Figure S19: NBO analysis across the reaction coordinates during the proton transfer step for **2**. Plot of the delocalization energy (E^2) between the donor LP(C1) and acceptor σ^*(Hw-Ow) NBOs. The standard deviations are shown as semi-transparent bands, while the inset image shows the extent to which the LP(C1) donor NBO is perturbed due to delocalization.

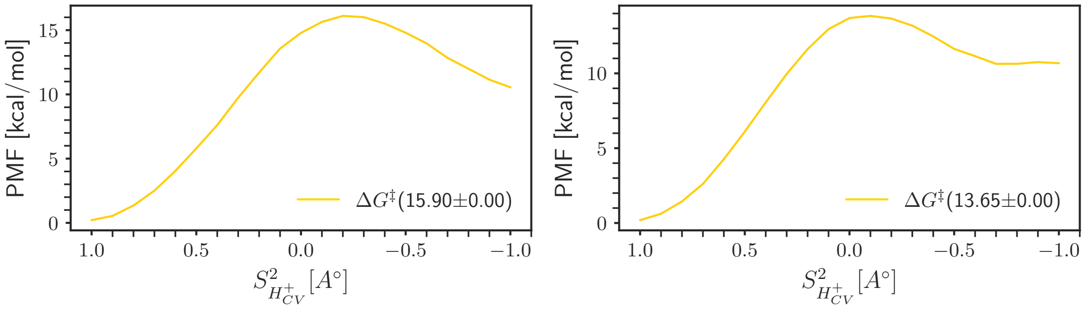

(a)

(b)

Figure S20: Potential of mean force (PMF) across the reaction coordinates during the proton transfer step for **2** via water (a), or via Y183 (b). Data were derived from 100 independent 1 ps QM/MM (B3LYP-6-31G*/amber19ffsb) SMD simulations. The mean square errors are shown as vertical bars.

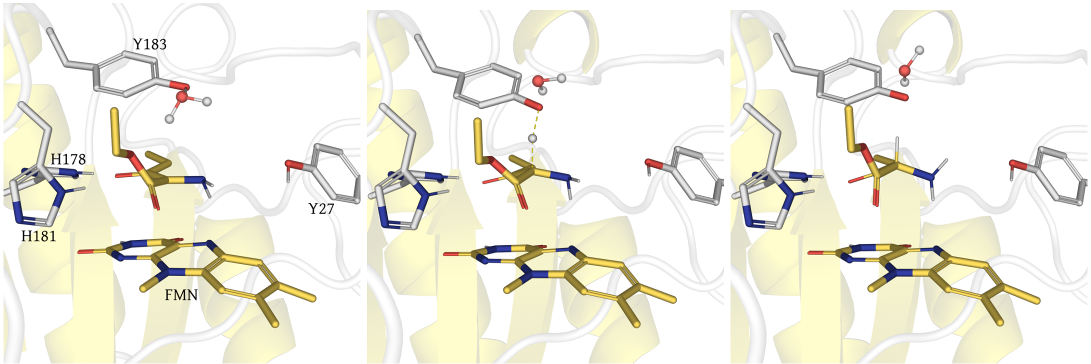

(a) Reactant State (RS^2_{H+}) (b) Transition State (TS^2_{H+}) (c) Product State (PS^2_{H+})

Figure S21: QM/MM (UB3LYP-6-31G*/OPLS2005) geometry optimised structures of the active site of XenA in the (RS^2_{H+}),(TS^2_{H+}), and (PS^2_{H+}) configurations with **2**. The configurations shown are the optimised molecular structures corresponding to stationary points along the lowest energy reaction coordinate for the direct proton transfer from Y183.

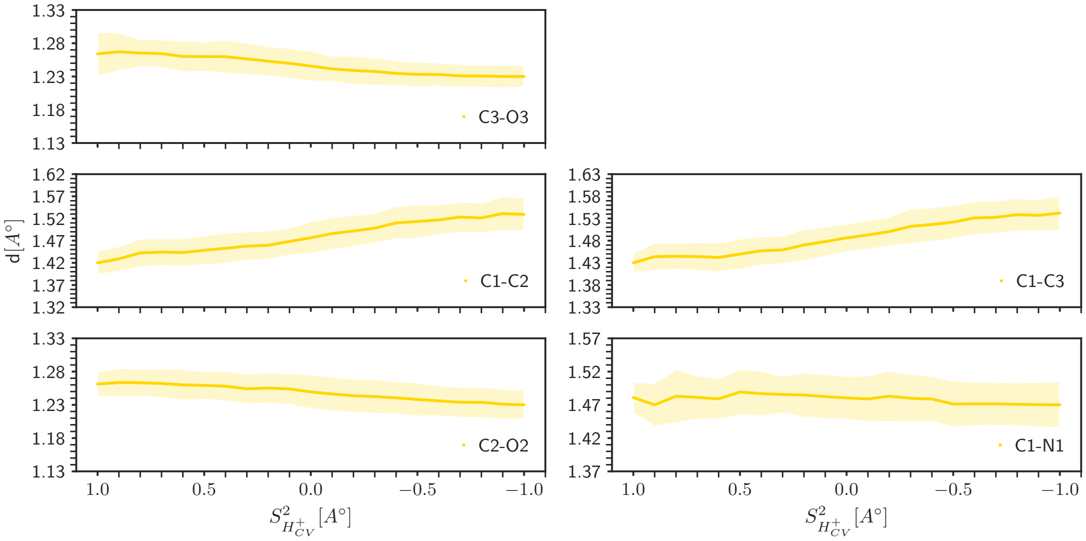

(a)

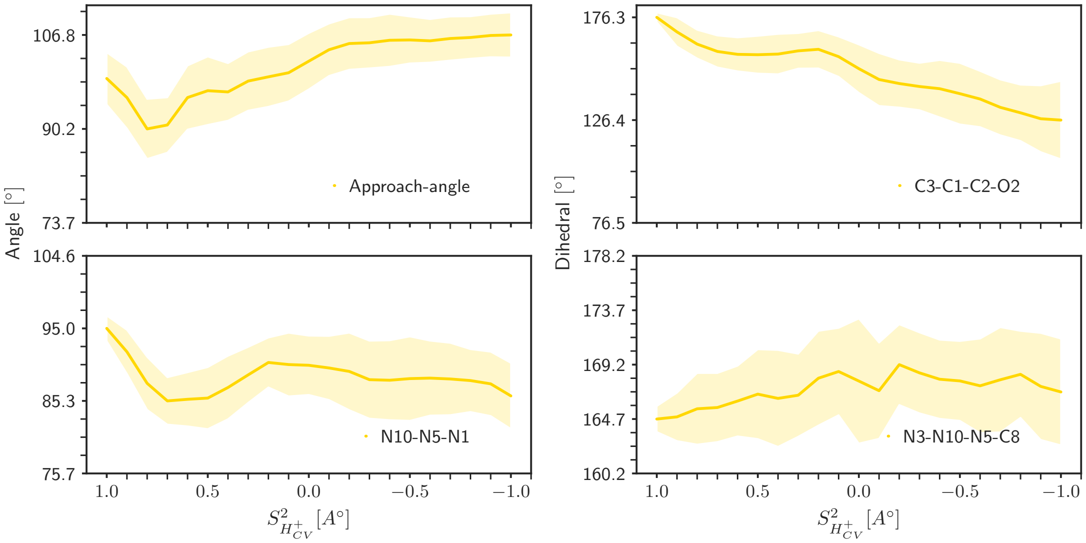

(b)

Figure S22: Dynamics of geometric changes across the reaction coordinates during the proton transfer step for **2**. (a) Intra-molecular distances within **2**, and (b) selected angles and dihedrals between **2** and XenA. The plots were generated using data from 100 independent 1 ps QM/MM (B3LYP-6-31G*/amber19ffsb) SMD simulations. The standard deviations are shown as semi-transparent bands.

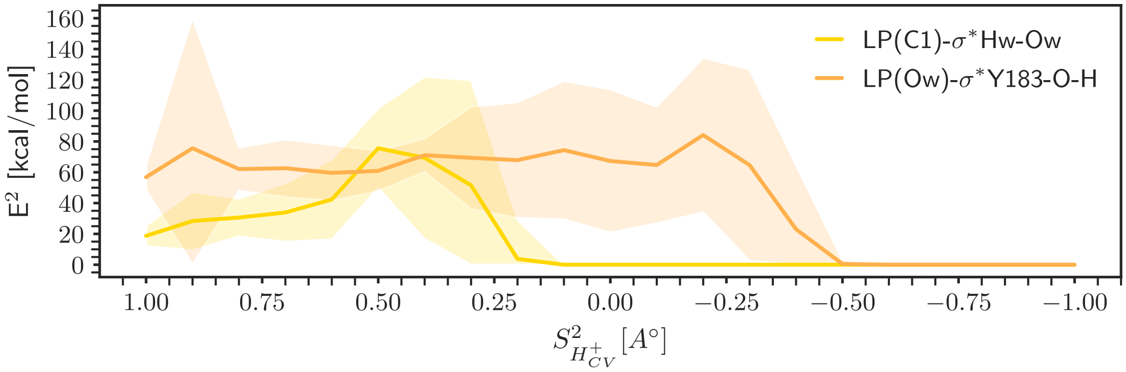

Figure S23: NBO analysis across the reaction coordinates during the proton transfer step for **2**. Plot of the delocalization energy (E^2) plot between the respective pairs of donor-acceptor NBOs. The standard deviations are shown as semi-transparent bands.

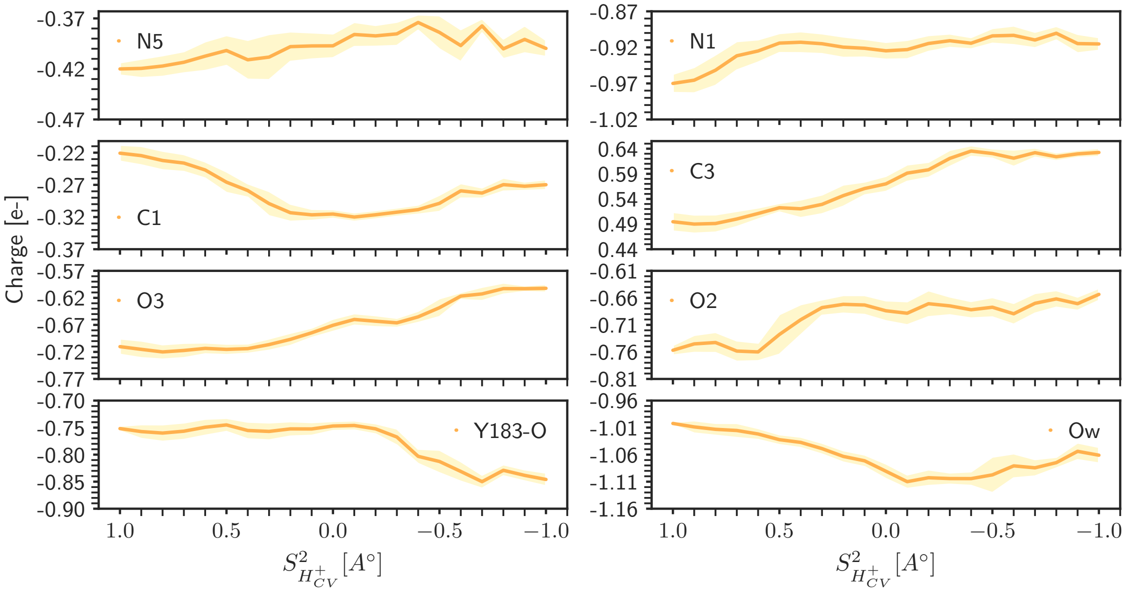

Figure S24: Dynamics of atom-centred charges across the reaction coordinates during the proton transfer step for **2**. The plots were generated using data obtained with the NBO program from 5 independent 1 ps QM/MM (B3LYP-6-31G*/amber19ffsb) SMD simulations. The standard deviations are shown as semi-transparent bands.

Detailed Methodology

Protein Structure Preparation

Complex of XenA and Oxime 1

The crystal structure (PDB-ID: 8AU8)[2] of XenA in complex with ethyl-(Z)-2-(hydroxyimino)-3-oxopentanoate (**1**) was used as the starting point for the calculations. In the crystal, XenA is a homodimer in which residue W358 of chain B interacts with the active site of chain A and *vice versa.*[3] Therefore, both chains were considered in this study. The protein structure was further prepared using the protein preparation wizard[4] in Maestro.[5] Specifically, the two active site histidines, H178 and H181, were modelled as neutral and singly protonated at the ϵ- and δ-nitrogen atoms, respectively. Our previous study suggested that the reduced enzyme would favour the neutral form of **1**; therefore, the hydroxylimino oxygen was modelled as protonated.[2] All crystal waters and ligands were removed except **1** and the flavin (FMN) cofactor. FMN was modelled in its negatively charged ($N1^-$) reduced state. Finally, the complex structure was subjected to an energy minimisation step in which only the hydrogen atoms were allowed to move.

Complex of XenA and Imine 2

The starting structure for the complex of XenA with ethyl-2-imino-3-oxopentanoate **2** resulted from the modelling calculations of the reductive dehydration of **1** as described in Scheme 1a in the main manuscript. FMN was reverted to its reduced state ($FMNH^-$), and this modelled complex was then used for further computations as follows.

Additionally, we have performed docking of **2** with XenA using DiffDock,[6] where we have generated 10 docking poses using the default settings of the DiffDock algorithm. We found that the orientation of the substrate **2** in the docked poses is also very similar to the binding pose of substrate **1** in the active site of XenA.

Molecular Simulations

Modeling Simulation System

After the preparatory steps listed above, the prepared flavoenzyme-substrate complex was used as input in the Amber20[7] program, where the complex was solvated in an octahedral box such that any enzyme atom should be at least 10 Å away from the box boundaries. The system was neutralised by adding the required number of Na^{+} or Cl^{-} ions. The molecular mechanics potential energy of the whole system was then derived using Amber ff19SB[8] for the protein, and its compatible version of the Amber general atom force-field[9] (GAFF2) for the ligands (flavin and substrate), the Joung and Cheatham parameters[10] for Na^{+}, and the SPCe[11] water model for water molecules.

Minimisation and Equilibration Using Molecular Mechanics (MM)

The system was then subjected to 2000 steps of energy minimisation using the steepest descent algorithm, and if this did not converge, followed by 2000 steps of conjugate gradient optimisation. Later, the temperature of the whole system was linearly increased from 0K to 298K for 20 ps, followed by a 10 ps NVT run at 298K. The volume of the system was then allowed to equilibrate during a 100 ps NPT run. During all these MM minimisation and equilibration steps, we kept a position restraint on each solute-heavy atom using a force constant of 10 kcal/mol/Å^2. Starting with a high-resolution crystal structure of the enzyme-substrate complex, our objective was to selectively optimise the positions of introduced hydrogen atoms and solvent molecules. Therefore, the chosen equilibration time was based on the recommended relaxation time specific to this context.[12] Also, periodic boundary conditions (PBC) were employed with bonds containing hydrogen atoms held rigid with SHAKE.[13] Temperature control was done with the Langevin thermostat,[14,15] and pressure control was achieved with the Berendsen barostat,[16] each with a relaxation time of 2.0 ps. A cutoff radius of 8 Å was used for non-bonded interactions. The MM simulations were

performed using a CUDA-enabled version of the program PMEMD.[17–19]

Minimisation Using Hybrid Method (QM/MM)

The system was then divided into two parts to employ the hybrid QM/MM Hamiltonian.[20] The atoms described in the QM3 region (Figure S25) were considered for the QM layer, whereas the remaining structure was treated by molecular mechanics. Subsequently, each system underwent further energy minimisation using the semi-empirical quantum mechanics/molecular mechanics method (SQM/MM), followed by the quantum mechanics/molecular mechanics (QM/MM) method without restraints. We have used the PM6-DH+[21,22] functional for SQM and the B3LYP[23–25] DFT functional with the 6-31G*[26] basis set for the QM caclulations. The electrostatic embedding scheme described in[27] was used to compute the interactions of MM point charges with the QM system. Hydrogen link atoms[28] were used to treat the QM/MM bonding interfaces. As we were dealing with hydride and proton transfer reactions, the SHAKE[13] algorithm was turned off for the QM atoms, which is otherwise used to constrain bonds involving hydrogen atoms.

QM Region

Selection of Residues for QM Region

One of the pertaining questions that arise when modelling enzymatic reactions using QM/MM is the composition of QM regions and which residues should be included. To address this particular question, we considered modelling different QM regions. We utilised the existing chemical information on XenA active site residues, such as the pair of histidine residues (H178 and H181) proposed to stabilise the delocalisation of charges on the substrate, Y183 as a potential proton donor, and Y27 forming H-bonds with the substrate. In addition, we incorporated active site water molecules in the QM region. Overall, we considered six different QM regions as depicted in detail in Figure S25 and summarised in Table S3. Starting from a model that consisted only of **1** and lumiflavin (LuF) (QM1), QM2 additionally has

two histidines (H178 and H181). The two tyrosine residues, Y27 and Y183, were incorporated in QM3, whereas we added one and two close water molecules in QM4 and QM5, respectively. Finally, QM6 was similar to QM4 but included the complete flavin ($FMNH^-$) instead of only LuF. At the beginning of each simulation, the residue ID of the nearest water molecule was identified and then explicitly specified for the respective QM region throughout the simulation.

Table S3: Summary of QM regions used in the QM/MM computations. Each QM region bears a net charge of -1, except QM6, which has a net charge of -3.

QM Regions	Residues	Total Atoms
QM1	oxime **1**, LuF[a]	55
QM2	QM1, H178[b], H181[b]	79
QM3	QM2, Y27[b], Y183[b]	111
QM4	QM3, 1Wat[c]	114
QM5	QM3, 2Wat[d]	117
QM6	QM4, $FMNH^-$ instead of LuF	133

[a] Lumiflavin. [b] SideChain Only. [c] Nearest water molecule. [d] 2nd nearest water molecule.

Selection of QM Method

Previous QM/MM investigations of flavoenzyme have employed B3LYP functional with a 6-31G* basis set for QM computations.[29–34] While B3LYP is the popularly used DFT functional for biological systems,[35] the inclusion of dispersion correction has been suggested to account for the missing London dispersion effect for long-range separations and solvated systems.[36–38] Our previous experimental investigation estimated that the activation energy ($\Delta E_a^\ddagger$) for hydride transfer to substrate **1** is 15.4 kcal/mol.[2] Considering that we have computed the $\Delta E_a^\ddagger$ by performing single-point energy calculations on UB3LYP/6-31G*//OPLS-2005 optimised structures of ($RS^1_{H^-}$), ($TS^1_{H^-}$) using atoms mentioned in QM4 region. First, we tested the B3LYP with different Gaussian basis sets without considering the dispersion correction term, and then we incorporated the D3[39] dispersion correction term; the computed $\Delta E_a^\ddagger$ is shown in Table S4. Adding the dispersion correction term to B3LYP has contributed around 1

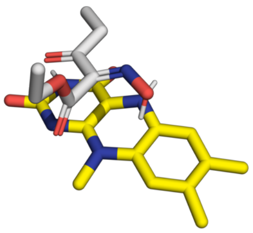

(a) QM1 [55 Atoms and Net Charge -1]

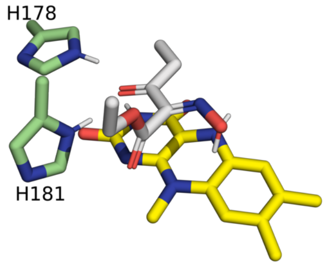

(b) QM2 [79 Atoms and Net Charge -1]

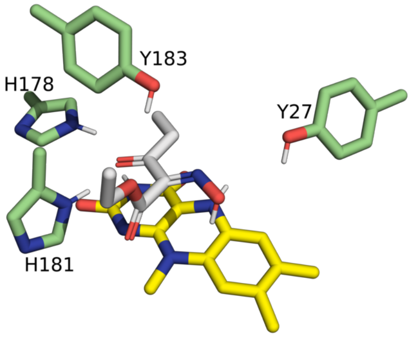

(c) QM3 [111 Atoms and Net Charge -1]

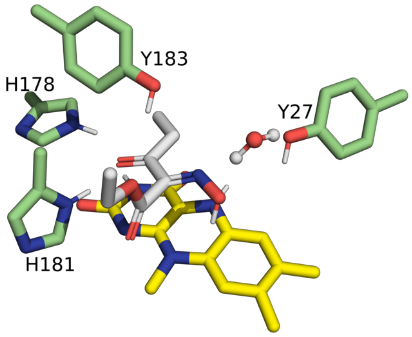

(d) QM4 [114 Atoms and Net Charge -1]

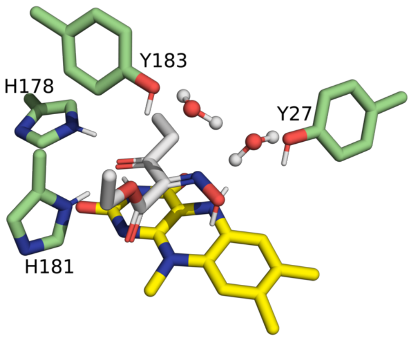

(e) QM5 [117 Atoms and Net Charge -1]

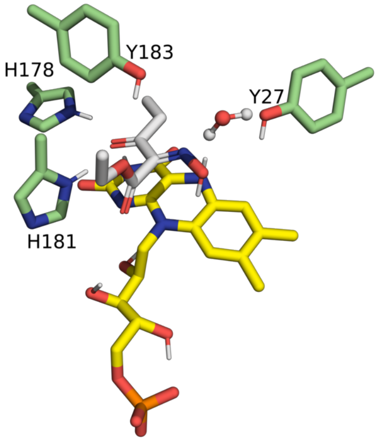

(f) QM6 [133 Atoms and Net Charge -3]

Figure S25: Atoms in the different QM regions: (a) QM1: lumiflavin (LuF) and **1**, (b) QM2: QM1 plus sidechains of H178 and H181, (c) QM3: QM2 plus sidechains of Y27 and Y183, (d) QM4: QM3 plus one closest water molecule, (e) QM5: QM4 plus the second closest water molecule, (f) QM6: the same as QM4 but with a complete flavin ($FMNH^-$) instead of LuF. The carbon atoms in LuF/$FMNH^-$ are shown in yellow, those of the substrate in grey, and those belonging to protein residues in green. Oxygen and nitrogen atoms are coloured red and blue, respectively. Polar hydrogen atoms are shown in white. Water molecules are shown in a ball-and-stick representations, while the remaining atoms are shown in stick representations.

kcal/mol more to the $\Delta E_a^\ddagger$ for each unique combination of B3LYP and Gaussian basis sets. However, adding more diffuse and/or polarization has lowered the $\Delta E_a^\ddagger$. We have also tested M06-2X density functional, which has been recommended for better accountability of non-covalent interactions across biological systems.[38,40] Indeed, the computed $\Delta E_a^\ddagger$ with M06-2X approaches the experimental $\Delta E_a^\ddagger$, especially, one with 6-31G+*. However, QM calculations with B3LYP are much faster than the M06 density functionals,[41] considering the higher computational cost for running hundreds of QM/MM MD simulations with M06-2X compared to B3LYP, we opt for B3LYP/6-31G* for running QM/MM MD simulations. Whereas, all the $\Delta E_a^\ddagger$ have been computed by performing single point energy computation with M06-2X/6-31G+*//OPLS-2005 on UB3LYP/6-31G*//OPLS-2005 optimised structures of stationary points of respective reaction coordinates. At the same time, the reported enthalpic changes for each reaction have been computed at the UB3LYP/6-31G*//OPLS-2005 level of theory due to expensive frequency calculations with M06-2X/6-31G+*//OPLS-2005.

Table S4: Benchmarking of QM methods to be used in the QM/MM computations.

QM Method	Basis Set	$\Delta E_a^\ddagger$ [kcal/mol]
B3LYP	6-31G*	8.04
	6-31G**	7.05
	6-31G+*	7.74
	6-31G+**	6.76
	6-31G++**	6.90
B3LYP-D3	6-31G*	9.02
	6-31G**	8.30
	6-31G+*	9.00
	6-31G+**	8.01
	6-31G++**	8.14
M06-2X	6-31G*	13.88
	6-31G**	12.91
	6-31G+*	14.19
	6-31G+**	13.25
	6-31G++**	13.41

$\Delta E_a^\ddagger$ = Computed activation energy for hydride transfer to substrate **1**.

Convergence of Partial Charge on Substrate

In the past, the convergence of the total partial charge on the substrate has been evaluated to choose an optimum number of QM atoms.[35,42] We have monitored this parameter for the substrate in the various QM regions and found that the difference between the total partial charge for the substrate converges as the number of atoms in the QM region increases (Figure S26). Interestingly, neither the number of water molecules significantly impacted the substrate's total partial charge nor the ribityl-phosphate tail of the flavin cofactor. Therefore, we used QM4 for all QM/MM computations. An issue of a zero or no gap between the highest-occupied and the lowest-unoccupied molecular orbitals (HOMO–LUMO) was reported during QM/MM simulations of proteins and solvated molecules.[42–44] However, we did not experience this gap-closing problem. All of our evaluated QM regions maintained a constant 2 eV HOMO-LUMO gap (Figure S26). For our subsequent investigations, we chose the B3LYP functional and a 6-31G* basis set for QM computation. Each of the above-described six QM regions underwent 5 independent 200 ps QM/MM simulations with a time step of 1 fs. The resulting 1000 frames of this 1 ps production run were used for both partial charge and HOMO-LUMO gap analyses.

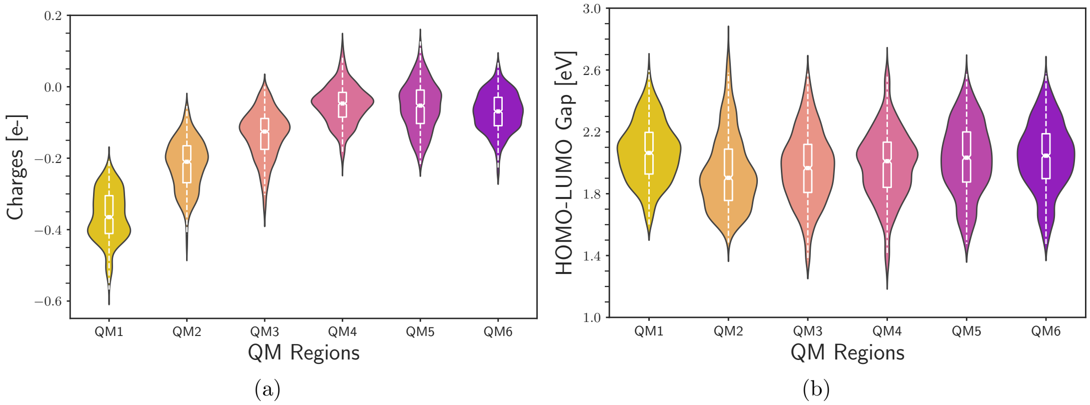

Figure S26: Violin plots showing (a) the sums of partial charges on **1** and (b) the HOMO-LUMO gaps when employing different QM regions. Charges and band gaps were computed using data obtained from 5 independent 200 ps QM/MM simulations.

QM/MM Production Run

For the production runs, we employed the Sander module of AMBER20[7] with Terachem[45,46] as an external QM package. As mentioned above, QM computations were done at the B3LYP/6-31G* level of theory, while the MM potential was derived from AMBER ff19SB.[8] Electrostatic embedding was employed to treat interactions between QM and MM regions, and a hydrogen link atom approach was used. The SHAKE[13] algorithm was turned off for all QM4 atoms in all subsequent QM/MM simulations. Interaction cutoffs, and thermostat and barostat parameters remained unchanged from the values given in the section (QM/MM Minimisation). Although we had included side chains of four protein residues in the QM4 region, we still needed to account for the fact that a protein residue outside of the defined QM region can affect ligand polarisation. We were using an electrostatic embedding scheme,[27] in which MM atoms that fall within the defined non-bonded interaction cutoff were considered as point charges, to model the electrostatic interactions with QM atoms and the polarisation of the QM electron density. Considering this, we have chosen the non-bonded cutoff of 8 Å, which is an optimum choice based on the findings of Willow et al.[47] Finally, using the QM/MM energy minimised structure as input, we run a continuous 5000 steps QM/MM MD run with a time-step of 1 fs, yielding a total simulation time of 5 ps. Snapshots of the trajectory were saved at each step.

Reaction Modeling

Computation of Reaction Coordinates

The whole 5 ps trajectory from the last step was loaded into VMD,[48] and the centre of the PBC box was re-aligned to the centre of mass of the protein. Solvent molecules not within 5 Å of any protein atom were removed, and the remaining atomic coordinates were saved as a PDB file. The generated PDB file was then opened in Maestero,[5] and input for Q-Site[49–51] (the QM/MM module of the Schrödinger package) was prepared by specifying the QM atoms

(atoms in the QM4 region), QM charge (-1), QM method and basis set (UB3LYP/6-31G*), and the MM potential (OPLS-2005[52]). Additionally, MM atoms more than 5 Å away from the QM region were fixed. Hydrogen atoms were used to cap covalent bonds partitioned at the QM/MM boundaries. Reactions were modelled by running a distance scan at a resolution of 0.1 Å against appropriately selected reaction coordinates, as depicted in Figure S27. From there, transition states were modelled using the quadratic synchronous transit (QST) method and further confirmed by the presence of a negative frequency corresponding to the selected reaction coordinates.

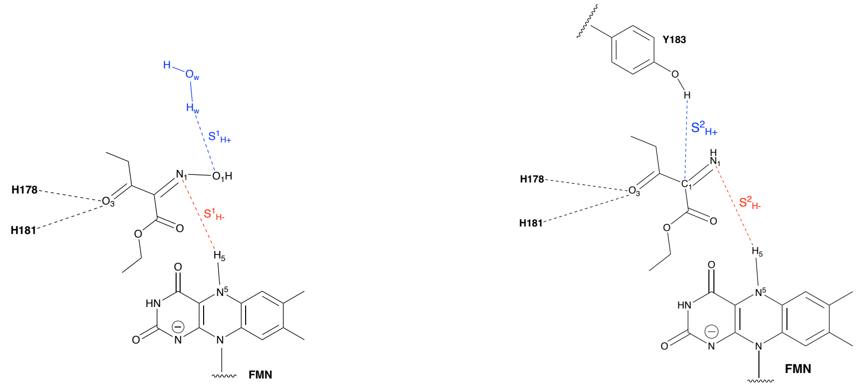

(a) (b)

Figure S27: Definition of reaction coordinates (collective variables, CVs) used in QM/MM scans for reaction modelling. (a) For **1**, $S1_{H-}$ is the hydride transfer reaction CV, and $S2_{H+}$ refers to a direct proton transfer from water. (b) For **2**, $S2_{H-}$ is the hydride transfer reaction CV, and $S2_{H+}$ refers to direct proton transfer from Y183. The depicted atoms were included in the QM/MM calculations. Dotted lines represent the reaction coordinates, and wavy lines depict the QM/MM boundary.

QM/MM Steered Molecular Dynamics

In addition to QM/MM geometry optimisation, we employed QM/MM Steered Molecular Dynamics (SMD) simulations to achieve a more dynamic and comprehensive understanding of the underlying reaction pathways. QM/MM SMD simulations facilitated a more rigorous

sampling of structural changes leading to a specific reaction coordinate, providing valuable information about the energy barriers and intermediates involved in the reaction. Using the reaction coordinates (CV) defined in Figure S28 for hydride and proton transfer, we performed 100 independent 1 ps SMD simulations for each CV. Out of the 100 independent QM/MM SMD runs, 5 runs were coupled with the Natural Bond Orbital (NBO)[53] program. Incorporating the NBO program with the QM/MM SMD simulations contributed significantly to a more detailed and precise analysis of the electronic changes during the chemical transformations. The velocity of pulling a hydrogen atom (a hydride or a proton, depending on the respective reaction coordinate) was 1 Å/ps, consistent with the best practices for QM/MM simulations of biological systems.[35] All simulation parameters remained the same as mentioned in the section (QM/MM Production Run). Finally, free energy changes along the defined CV were calculated by the fluctuation-dissipation (FD) estimator (see equation 1).[54,55] Mean square errors (MSE) as suggested by Gore *et al.* were computed according to equation 2 in order to assess the quality of the free energy estimator.[56]

$$\Delta F_{\gamma} = \langle W(\gamma) \rangle - \frac{\beta}{2}(\sigma_W^2) \tag{1}$$

$$MSE_{FD} = \frac{\sigma_W^2}{N} + \frac{\sigma_W^4}{2(N-1)} \tag{2}$$

In the above equations, γ denotes the reaction coordinate, and N is the total number of samples. β is equal to the Boltzmann constant (k_B)*(Temperature = 298K), $\langle W(\gamma) \rangle$ and σ^2 are the work average and the variance of the work distribution for a particular value of γ, respectively.

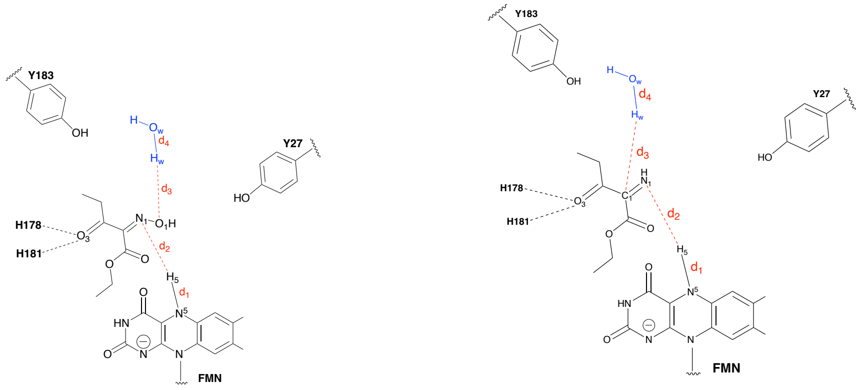

(a) (b)

Figure S28: Definition of reaction coordinates (collective variables, CVs) used in the QM/MM SMD simulation for (a) **1** and (b) **2**. Both CVs are linear combinations of distances (LCOD). The values of the hydride (H^{-}_{CV}) and proton transfer (H^{+}_{CV}) CVs are (d_1 - d_2) and (d_3 - d_4), respectively. The depicted atoms were included in the QM/MM calculations. Dotted lines represent reaction coordinates, and wavy lines depict the QM/MM boundary.

Tutorial

In addition, we have a publicly available detailed tutorial explaining the whole methodology discussed here on GitHub (https://github.com/hopanoid/Enzyme-Reaction-Dynamics-Tutorial).

References

(1) PyMOL Molecular Graphics System, version 2.0. Schrödinger, LLC, 2022.

(2) Breukelaar, W. B.; Polidori, N.; Singh, A.; Daniel, B.; Glueck, S. M.; Gruber, K.; Kroutil, W. Mechanistic Insights into the Ene-Reductase-Catalyzed Promiscuous Reduction of Oximes to Amines. *ACS Catalysis* **2023**, *13*, 2610–2618.

(3) Spiegelhauer, O.; Werther, T.; Mende, S.; Knauer, S. H.; Dobbek, H. Determinants of substrate binding and protonation in the flavoenzyme xenobiotic reductase A. *Journal of Molecular Biology* **2010**, *403*, 286–298.

(4) Madhavi Sastry, G.; Adzhigirey, M.; Day, T.; Annabhimoju, R.; Sherman, W. Protein and ligand preparation: parameters, protocols, and influence on virtual screening enrichments. *Journal of Computer-Aided Molecular Design* **2013**, *27*, 221–234.

(5) Maestero, version 2022.1. Schrödinger, LLC, 2022.

(6) Corso, G.; Stärk, H.; Jing, B.; Barzilay, R.; Jaakkola, T. Diffdock: Diffusion steps, twists, and turns for molecular docking. *arXiv preprint arXiv:2210.01776* **2022**,

(7) Case, D. et al. AMBER, version 20. University of California, San Francisco, 2020.

(8) Tian, C.; Kasavajhala, K.; Belfon, K. A.; Raguette, L.; Huang, H.; Migues, A. N.; Bickel, J.; Wang, Y.; Pincay, J.; Wu, Q., et al. ff19SB: Amino-acid-specific protein backbone parameters trained against quantum mechanics energy surfaces in solution. *Journal of Chemical Theory and Computation* **2019**, *16*, 528–552.

(9) Wang, J.; Wolf, R. M.; Caldwell, J. W.; Kollman, P. A.; Case, D. A. Development and testing of a general amber force field. *Journal of Computational Chemistry* **2004**, *25*, 1157–1174.

(10) Joung, I. S.; Cheatham III, T. E. Determination of alkali and halide monovalent ion parameters for use in explicitly solvated biomolecular simulations. *The Journal of Physical Chemistry B* **2008**, *112*, 9020–9041.

(11) HJC, B.; JR, G.; TP, S. The missing term in effective pair potentials. *The Journal of Physical Chemistry* **1987**, *91*, 6269–6271.

(12) Bizzarri, A. R.; Cannistraro, S. Molecular dynamics of water at the protein- solvent interface. *The Journal of Physical Chemistry B* **2002**, *106*, 6617–6633.

(13) Ryckaert, J.-P.; Ciccotti, G.; Berendsen, H. J. Numerical integration of the cartesian equations of motion of a system with constraints: molecular dynamics of n-alkanes. *Journal of Computational Physics* **1977**, *23*, 327–341.

(14) Schneider, T.; Stoll, E. Molecular-dynamics study of a three-dimensional one-component model for distortive phase transitions. *Physical Review B* **1978**, *17*, 1302.

(15) Bussi, G.; Parrinello, M. Accurate sampling using Langevin dynamics. *Physical Review E* **2007**, *75*, 056707.

(16) Berendsen, H. J.; Postma, J. v.; Van Gunsteren, W. F.; DiNola, A.; Haak, J. R. Molecular dynamics with coupling to an external bath. *The Journal of Chemical Physics* **1984**, *81*, 3684–3690.

(17) Salomon-Ferrer, R.; Gotz, A. W.; Poole, D.; Le Grand, S.; Walker, R. C. Routine microsecond molecular dynamics simulations with AMBER on GPUs. 2. Explicit solvent particle mesh Ewald. *Journal of Chemical Theory and Computation* **2013**, *9*, 3878–3888.

(18) Gotz, A. W.; Williamson, M. J.; Xu, D.; Poole, D.; Le Grand, S.; Walker, R. C. Routine microsecond molecular dynamics simulations with AMBER on GPUs. 1. Generalized born. *Journal of Chemical Theory and Computation* **2012**, *8*, 1542–1555.

(19) Le Grand, S.; Götz, A. W.; Walker, R. C. SPFP: Speed without compromise—A mixed precision model for GPU accelerated molecular dynamics simulations. *Computer Physics Communications* **2013**, *184*, 374–380.

(20) Bentzien, J.; Muller, R. P.; Florián, J.; Warshel, A. Hybrid ab initio quantum mechanics/molecular mechanics calculations of free energy surfaces for enzymatic reactions: the nucleophilic attack in subtilisin. *The Journal of Physical Chemistry B* **1998**, *102*, 2293–2301.

(21) Stewart, J. J. Optimization of parameters for semiempirical methods V: Modification of NDDO approximations and application to 70 elements. *Journal of Molecular Modeling* **2007**, *13*, 1173–1213.

(22) Korth, M. Third-generation hydrogen-bonding corrections for semiempirical QM methods and force fields. *Journal of Chemical Theory and Computation* **2010**, *6*, 3808–3816.

(23) Becke, A. D. A new mixing of Hartree–Fock and local density-functional theories. *The Journal of Chemical Physics* **1993**, *98*, 1372–1377.

(24) Becke, A. D. Density-functional exchange-energy approximation with correct asymptotic behavior. *Physical Review A* **1988**, *38*, 3098.

(25) Lee, C.; Yang, W.; Parr, R. G. Development of the Colle-Salvetti correlation-energy formula into a functional of the electron density. *Physical Review B* **1988**, *37*, 785.

(26) Hariharan, P. C.; Pople, J. A. The influence of polarization functions on molecular orbital hydrogenation energies. *Theoretica Chimica Acta* **1973**, *28*, 213–222.

(27) Nam, K.; Gao, J.; York, D. M. An efficient linear-scaling Ewald method for long-range electrostatic interactions in combined QM/MM calculations. *Journal of Chemical Theory and Computation* **2005**, *1*, 2–13.

(28) Field, M. J.; Bash, P. A.; Karplus, M. A combined quantum mechanical and molecular mechanical potential for molecular dynamics simulations. *Journal of Computational Chemistry* **1990**, *11*, 700–733.

(29) Lonsdale, R.; Reetz, M. T. Reduction of α, β-unsaturated ketones by old yellow enzymes: Mechanistic insights from quantum mechanics/molecular mechanics calculations. *Journal of the American Chemical Society* **2015**, *137*, 14733–14742.

(30) Capone, M.; Dell'Orletta, G.; Nicholls, B. T.; Scholes, G. D.; Hyster, T. K.; Aschi, M.; Daidone, I. Evidence of a Distinctive Enantioselective Binding Mode for the Photoinduced Radical Cyclization of α-Chloroamides in Ene-Reductases. *ACS Catalysis* **2023**, *13*, 15310–15321.

(31) Zhao, B.; Feng, J.; Yu, L.; Xing, Z.; Chen, B.; Liu, A.; Liu, F.; Shi, F.; Zhao, Y.; Tian, C., et al. Direct visible-light-excited flavoproteins for redox-neutral asymmetric radical hydroarylation. *Nature Catalysis* **2023**, 1–9.

(32) Sattelle, B. M. *Towards Tuning Redox Potentials: Theoretical Studies of Flavoproteins*; The University of Manchester (United Kingdom), 2008.

(33) Messiha, H. L.; Bruce, N. C.; Sattelle, B. M.; Sutcliffe, M. J.; Munro, A. W.; Scrutton, N. S. Role of active site residues and solvent in proton transfer and the modulation of flavin reduction potential in bacterial morphinone reductase. *Journal of Biological Chemistry* **2005**, *280*, 27103–27110.

(34) Delgado, M.; Gorlich, S.; Longbotham, J. E.; Scrutton, N. S.; Hay, S.; Moliner, V.; Tuñón, I. Convergence of theory and experiment on the role of preorganization, quan-

tum tunneling, and enzyme motions into flavoenzyme-catalyzed hydride transfer. *ACS catalysis* **2017**, *7*, 3190–3198.

(35) Clemente, C. M.; Capece, L.; Martí, M. A. Best Practices on QM/MM Simulations of Biological Systems. *Journal of Chemical Information and Modeling* **2023**, *63*, 2609–2627.

(36) Kruse, H.; Goerigk, L.; Grimme, S. Why the standard B3LYP/6-31G* model chemistry should not be used in DFT calculations of molecular thermochemistry: understanding and correcting the problem. *The Journal of organic chemistry* **2012**, *77*, 10824–10834.

(37) Lonsdale, R.; Harvey, J. N.; Mulholland, A. J. Effects of dispersion in density functional based quantum mechanical/molecular mechanical calculations on cytochrome P450 catalyzed reactions. *Journal of Chemical Theory and Computation* **2012**, *8*, 4637–4645.

(38) Goerigk, L.; Grimme, S. A thorough benchmark of density functional methods for general main group thermochemistry, kinetics, and noncovalent interactions. *Physical Chemistry Chemical Physics* **2011**, *13*, 6670–6688.

(39) Grimme, S.; Antony, J.; Ehrlich, S.; Krieg, H. A consistent and accurate ab initio parametrization of density functional dispersion correction (DFT-D) for the 94 elements H-Pu. *The Journal of chemical physics* **2010**, *132*.

(40) Zhao, Y.; Truhlar, D. G. The M06 suite of density functionals for main group thermochemistry, thermochemical kinetics, noncovalent interactions, excited states, and transition elements: two new functionals and systematic testing of four M06-class functionals and 12 other functionals. *Theoretical chemistry accounts* **2008**, *120*, 215–241.

(41) Rommel, J. B.; Kastner, J. The fragmentation–recombination mechanism of the enzyme glutamate mutase studied by QM/MM simulations. *Journal of the American Chemical Society* **2011**, *133*, 10195–10203.

(42) Kulik, H. J.; Zhang, J.; Klinman, J. P.; Martínez, T. J. How large should the QM region be in QM/MM calculations? The case of catechol O-methyltransferase. *The Journal of Physical Chemistry B* **2016**, *120*, 11381–11394.

(43) Lever, G.; Cole, D. J.; Hine, N. D.; Haynes, P. D.; Payne, M. C. Electrostatic considerations affecting the calculated HOMO–LUMO gap in protein molecules. *Journal of Physics: Condensed Matter* **2013**, *25*, 152101.

(44) Rudberg, E. Difficulties in applying pure Kohn–Sham density functional theory electronic structure methods to protein molecules. *Journal of Physics: Condensed Matter* **2012**, *24*, 072202.

(45) Ufimtsev, I. S.; Martinez, T. J. TeraChem. PetaChem, LLC, 2021.

(46) Ufimtsev, I. S.; Martinez, T. J. Quantum chemistry on graphical processing units. 1. Strategies for two-electron integral evaluation. *Journal of Chemical Theory and Computation* **2008**, *4*, 222–231.

(47) Willow, S. Y.; Xie, B.; Lawrence, J.; Eisenberg, R. S.; Minh, D. D. On the polarization of ligands by proteins. *Physical Chemistry Chemical Physics* **2020**, *22*, 12044–12057.

(48) Humphrey, W.; Dalke, A.; Schulten, K. VMD: visual molecular dynamics. *Journal of Molecular Graphics* **1996**, *14*, 33–38.

(49) Qsite, version 2022.1. Schrödinger, LLC, 2022.

(50) Philipp, D. M.; Friesner, R. A. Mixed ab initio QM/MM modeling using frozen orbitals and tests with alanine dipeptide and tetrapeptide. *Journal of Computational Chemistry* **1999**, *20*, 1468–1494.

(51) Murphy, R. B.; Philipp, D. M.; Friesner, R. A. A mixed quantum mechanics/molecular mechanics (QM/MM) method for large-scale modeling of chemistry in protein environments. *Journal of Computational Chemistry* **2000**, *21*, 1442–1457.

(52) Shivakumar, D.; Harder, E.; Damm, W.; Friesner, R. A.; Sherman, W. Improving the prediction of absolute solvation free energies using the next generation OPLS force field. *Journal of Chemical Theory and Computation* **2012**, *8*, 2553–2558.

(53) Glendening, E. D.; Badenhoop, J. K.; Reed, A. E.; Carpenter, J. E.; Bohmann, J. A.; Morales, C. M.; Karafiloglou, P.; Landis, C. R.; Weinhold, F. NBO, version 7.0. University of Wisconsin, Madison, 2018.

(54) Callen, H. B.; Welton, T. A. Irreversibility and generalized noise. *Physical Review* **1951**, *83*, 34.

(55) Arrar, M.; Boubeta, F. M.; Szretter, M. E.; Sued, M.; Boechi, L.; Rodriguez, D. On the accurate estimation of free energies using the Jarzynski equality. *Journal of Computational Chemistry* **2019**, *40*, 688–696.

(56) Gore, J.; Ritort, F.; Bustamante, C. Bias and error in estimates of equilibrium free-energy differences from nonequilibrium measurements. *Proceedings of the National Academy of Sciences* **2003**, *100*, 12564–12569.
